# Colossal Dielectric Perovskites of Calcium Copper Titanate (CaCu_3_Ti_4_O_12_) with Low‐Iridium Dopants Enables Ultrahigh Mass Activity for the Acidic Oxygen Evolution Reaction

**DOI:** 10.1002/advs.202207695

**Published:** 2023-03-29

**Authors:** Nguyen Thi Thu Thao, Kwangsoo Kim, Jeong Ho Ryu, Byeong‐Seon An, Arpan Kumar Nayak, Jin Uk Jang, Kyeong‐Han Na, Won‐Youl Choi, Ghulam Ali, Keun Hwa Chae, Muhammad Akbar, Kyung Yoon Chung, Hyun‐Seok Cho, Jong Hyeok Park, Byung‐Hyun Kim, HyukSu Han

**Affiliations:** ^1^ Department of Energy Engineering Konkuk University 05029 120 Neungdong‐ro Seoul Republic of Korea; ^2^ Computational Science & Engineering Laboratory Korea Institute of Energy Research 34129 152 Gajeong‐ro, Yuseong‐gu Daejeon Republic of Korea; ^3^ Department of Chemical and Biomolecular Engineering Yonsei University 03722 50 Yonsei‐ro Seoul Republic of Korea; ^4^ Department of Materials Science and Engineering Korea National University of Transportation 27469 50 Daehak‐ro Chungju Republic of Korea; ^5^ Analysis Center for Energy Research Korea Institute of Energy Research 34129 152 Gajeong‐ro, Yuseong‐gu Daejeon Republic of Korea; ^6^ Department of Metal and Materials Engineering, Gangneung‐Wonju National University 25457 7 Jukheongil, Gangneung Gangwon Republic of Korea; ^7^ Smart Hydrogen Energy Center Gangneung‐Wonju National University 25457 7 Jukheongil, Gangneung Gangwon Republic of Korea; ^8^ U.S.‐Pakistan Center for Advanced Studies in Energy (USPCASE) National University of Sciences and Technology (NUST) H‐12 Islamabad Pakistan; ^9^ Advanced Analysis Center Korea Institute of Science and Technology 02792 Hwarang‐ro 14‐gil 5, Seongbuk‐gu Seoul Republic of Korea; ^10^ Energy Storage Research Center Korea Institute of Science and Technology 02792 Hwarang‐ro 14‐gil 5, Seongbuk‐gu Seoul Republic of Korea; ^11^ Division of Energy and Environment Technology KIST School Korea University of Science and Technology 02792 Seoul Republic of Korea; ^12^ Hydrogen Research Department Korea Institute of Energy Research 34129 152 Gajeong‐ro, Yuseong‐gu Daejeon Republic of Korea; ^13^ Computational Science & Engineering Laboratory Korea Institute of Energy Research 152 Gajeong‐ro, Yuseong‐gu Daejeon 34129 Republic of Korea

**Keywords:** calcium copper titanate, electrocatalysts, low iridium, oxygen evolution reaction

## Abstract

Oxygen evolution reaction (OER) under acidic conditions becomes of significant importance for the practical use of a proton exchange membrane (PEM) water electrolyzer. In particular, maximizing the mass activity of iridium (Ir) is one of the maiden issues. Herein, the authors discover that the Ir‐doped calcium copper titanate (CaCu₃Ti₄O₁₂, CCTO) perovskite exhibits ultrahigh mass activity up to 1000 A g_Ir_^−1^ for the acidic OER, which is 66 times higher than that of the benchmark catalyst, IrO_2_. By substituting Ti with Ir in CCTO, metal‐oxygen (M‐O) covalency can be significantly increased leading to the reduced energy barrier for charge transfer. Further, highly polarizable CCTO perovskite referred to as “colossal dielectric”, possesses low defect formation energy for oxygen vacancy inducing a high number of oxygen vacancies in Ir‐doped CCTO (Ir‐CCTO). Electron transfer occurs from the oxygen vacancies and Ti to the substituted Ir consequentially resulting in the electron‐rich Ir and ‐deficient Ti sites. Thus, favorable adsorptions of oxygen intermediates can take place at Ti sites while the Ir ensures efficient charge supplies during OER, taking a top position of the volcano plot. Simultaneously, the introduced Ir dopants form nanoclusters at the surface of Ir‐CCTO, which can boost catalytic activity for the acidic OER.

## Graphical abstract

The use of a colossal dielectric material (CaCu₃Ti₄O₁₂, CCTO) as an effective promoter for iridium (Ir) dopants can be a new and revolutionary catalyst design concept for realizing Ir‐based electrocatalyst with an exceptionally high mass activity for the acidic oxygen evolution reaction (OER). The Ir‐incorporated CCTO (Ir‐CCTO) takes a position on top of the OER volcano plot satisfying the Sabatier principles.

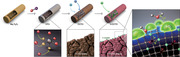

## 1 Introduction

Water oxidation is key to the electrochemical process associated with important energy conversion devices, such as water electrolyzers.^[^ ^1^, ^2^, ^3^, ^4^, ^5^, ^6^ ^]^ In water electrolyzers, high overpotential is always required at the anode where the OER occurs due to the four‐electron/four‐proton‐involved sluggish reaction kinetics.^[^ ^7^, ^8^, ^9^ ^]^ This results in considerable energy losses. Hence, expediting the OER process using suitable electrocatalysts with reduced overpotential is an urgent demand for advanced electrochemical devices to increase overall efficiency.

A number of experimental and theoretical studies have been conducted to develop electrocatalysts that can ensure efficient OER.^[^ ^5^, ^10^ ^]^ Non‐noble metal electrocatalysts, including iron, nickel, and cobalt‐based bi‐ or multi‐metallic (hydro)oxides in amorphous or crystalline phases, have successfully demonstrated their high electrocatalytic activity and stability under alkaline conditions, even surpassing that of iridium or ruthenium dioxides (IrO_2_ or RuO_2_).^[^ ^11^, ^12^, ^13^ ^]^ However, all these catalysts are unstable under OER conditions in acidic media, and currently only Ir‐based catalysts have shown promising activity and stability for acidic OER. Considering the urgent demands for acidic OER and the scarcity of Ir (0.001 ppm in the Earth's crust),^[^ ^14^ ^]^ the development of highly active and durable electrocatalysts with ultrahigh Ir‐mass activity for acidic OER is indeed indispensable but challenging.

Although recent efforts have been made to develop low‐iridium materials for acidic OER, their success is quite limited. More critically, the iridium content in the materials was often too high to secure sufficient Ir‐mass activity, which is the most important factor for practical applications of acidic OER. In addition, obtaining good durability of electrocatalysts without significant surface reconstruction during acidic OER is another challenging issue.^[^ ^15^, ^16^, ^17^, ^18^, ^19^ ^]^ The electrocatalytic activity and stability of Ir‐based metal oxide catalysts are strongly dependent on the material properties of metal oxide supports in which the Ir is incorporated either as substitutional atomic dopants or anchored oxidative nanoclusters.^[^ ^20^, ^21^, ^22^ ^]^ Importantly, in either case, facile electronic transfers between the active Ir/Ir‐O_x_ sites and the metal oxide supports as well as a favorable adsorption energy landscape for reaction intermediates are crucial to boosting iridium mass activity for OER under acidic conditions.

Calcium copper titanate, CCTO (space group: Im3, No. 204), is one of the perovskite types of multi‐metallic oxides exhibiting extraordinarily high polarizability associated with a large number of space charges in the grains, referred to as “colossal permittivity” (*ε*_r_ higher than 10 000).^[^ ^23^, ^24^ ^]^ For CCTO, its high polarizability may be advantageous for the adsorptions of reactants or intermediates in the electrolyte, and at the same time, the high concentration of space charges or point defects (i.e., oxygen vacancy) in the grains can promote ionic or electronic charge transfers during electrolysis. Additionally, Ti^4+^ ions in the CCTO lattice have a similar ionic radius to that of Ir^4+^ (only a 3.27% difference), satisfying the Hume–Rothery rules suitable for the formation of substitutional solid solutions. When Ir substitutes Ti sites in the CCTO perovskite structure, charge exchanges will readily occur within neighboring transition metals and existing point defects due to different electron affinities, leading to tuned surface electronic configurations. This may offer a high possibility for available active sites with optimized adsorption energies for the reactants. Moreover, perovskite materials have been intermittently prepared in micrometer‐sized particles or thin films, possibly limiting the number of accessible catalytic active sites due to low surface area.^[^ ^24^, ^25^, ^26^, ^27^ ^]^ In this regard, the synthesis and use of nanostructured CCTO as a metal oxide support for incorporating iridium active sites are of significant importance in our pursuit to design Ir‐based electrocatalysts with enhanced iridium mass activity and stability for acidic OER.

Herein, we disclose that the incorporation of Ir into colossal dielectric CCTO perovskite enables ultrahigh mass activity up to 1000 A g_Ir_^−1^ for the acidic OER. We demonstrate that the incorporated Ir simultaneously acts as a substitutional dopant for Ti sites in the perovskite lattice and an atomic seed for the nucleation of Ir‐nanoclusters at the CCTO surface. The substitutional doping of Ir creates a high number of oxygen vacancies in the CCTO lattice, owing to its structural and electronic flexibility. It is clearly addressed from the atomic‐scale microscopic and spectroscopic measurements, in combination with density functional theory (DFT) calculations, that the M‐O covalency can be substantially increased by incorporating Ir into CCTO, reducing the charge–transfer energy and thus enhancing OER activity. This also plays a decisive factor in securing favorable adsorption thermodynamics of Ir‐CCTO for acidic OER, taking the top position of the OER volcano plot by satisfying Sabatier principles. As a consequence, Ir‐CCTO exhibits excellent catalytic activity and durability with ultrahigh mass activity, 66 times higher than that of the benchmark IrO_2_ catalyst. To our knowledge, the use of colossal dielectric materials for acidic OER has never been attempted, and furthermore, such a high mass activity up to 1000 A g_Ir_^−1^ has indeed rarely been reported.

## 2 Results and Discussion

**Figure 1:**
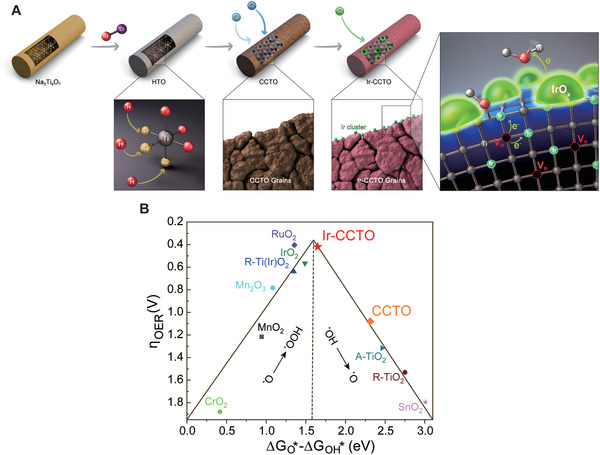
A) Schematic illustration of catalyst design strategy for Ir‐CCTO NBs. B) Calculated volcano plot presenting the theoretical overpotentials of OER as a function of $\Delta G_{O^{*}}-\Delta G_{OH^{*}}$ values of different catalysts including CCTO and Ir‐CCTO.

The catalyst design strategy used in this work is illustrated in **Figure** **1**A. Briefly, hydrogenated titanate (H_2_Ti_3_O_7_, HTO, space group: C2/m) was used as a starting material to prepare the Ir‐doped CCTO nanobelts (Ir‐CCTO NBs). HTO was synthesized via a facile hydrothermal reaction with a subsequent ionic exchange method (see the Experimental Section in Supporting Information). The as‐prepared HTO was transformed into CCTO and Ir‐CCTO with subsequent reactions. The introduced Ir dopants were incorporated into CCTO in two different directions: on the one hand, Ir was atomically doped into the semiconductive CCTO grains; on the other hand, Ir^3+^ ions formed IrO*_x_* nanoclusters at the surface of CCTO nanobelts. The atomically doped Ir will generate more defects, such as oxygen vacancies, in the perovskite lattice of CCTO, while the IrO*_x_* will offer active reaction sites for OER under acidic conditions. In Ir‐CCTO, we hypothesize that electron transfers are likely to occur among the generated defects and the incorporated Ir dopants and the surface IrO*_x_* nanoclusters, resulting in both electron‐rich Ir site/clusters and electron‐deficient transition metal sites (i.e., Ti or Cu) due to the electronegativity difference. This surface electronic state accelerates water dissociation and adsorption steps during OER and subsequent charge transport from the adsorbents to the surface, thus boosting the catalytic activity for OER. Surprisingly, our density functional theory (DFT) calculations revealed that the Ir‐CCTO is positioned at the top of OER volcano plot implying its high catalytic activity (Figure 1B). This result will be revisited at the end after investigating physical and chemical modulations in the Ir‐CCTO leading to the outstanding catalytic performance in terms of atomic‐scale material engineering aspects.

**Figure 2:**
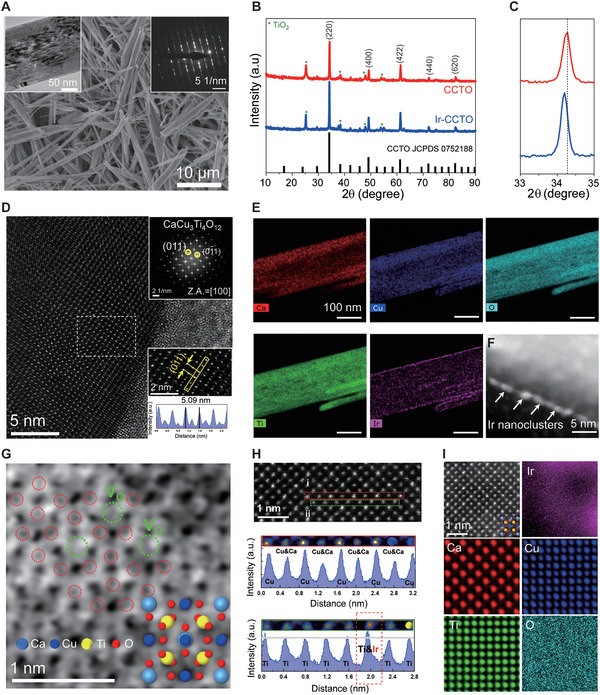
Materials characterization for Ir‐CCTO NBs. A) SEM image of as‐preapred HTO NBs. The left inset is a TEM image of Ir‐CCTO NBs, and the right inset shows the corresponding SAED. B) Powder XRD pattern of CCTO and Ir‐CCTO NBs. C) Enlarged (220) diffraction peak showing slight negative peak shift for Ir‐CCTO NBs. D) HR‐TEM image of Ir‐CCTO NBs. The upper inset shows the corresponding SAED taken at a [100] zone axis. The lower inset shows the interplanar distance of the marked region (white rectangular) in (C). E) STEM‐EDX elemental mapping of Ir‐CCTO NBs for Ca, Cu, O, Ti, and Ir. F) Dark field‐STEM image of Ir‐CCTO NBs. G) Annular bright field‐STEM image of Ir‐CCTO NBs with the simulated atomic structure of CCTO perovskite. H) High angle annular dark field‐STEM (HAADF‐STEM) image with line profiles for A and B‐sites atomic intensity at marked regions (red and green rectangular). I) Atomic‐scale STEM‐EDX mapping images for Ir, Ca, Cu, Ti, and O in Ir‐CCTO NBs.

As‐prepared Ir‐CCTO showed a well‐defined 1D nanobelt morphology with a predominant diameter in the range of 200–300 nm (**Figure** **2**A and Figure S1, Supporting Information, see Experimental Section for experimental details). Here, CCTO nanobelts calcined at 600 °C were used for preparing Ir‐CCTO owing to its morphology and phase (Figures S1 and S2A, Supporting Information). A selected‐area electron diffraction (SAED) pattern using transmission electron microscopy (TEM) revealed the high crystalline nature of Ir‐CCTO NBs (the right upper inset of Figure 2A). Note that Ir‐CCTO NBs synthesized using the Ir:Ti atomic ratio of 0.05, a reaction temperature of 150 °C, and a reaction time of 24 h showed the best electrocatalytic activity for OER under acidic conditions while preserving good nanostructure (Figures S3 and S4, Supporting Information). Therefore, the following results and discussions are focused on this material unless specifically mentioned. Powder X‐ray diffraction (PXRD) patterns revealed that Ir‐CCTO NBs were crystallized in the perovskite crystal, which was identical to the pristine CCTO NBs (Figure 2B). Note that the low intense peaks at *≈*38°, ≈48°, and ≈55° are well matched (Figure 2B and Figure S2A, Supporting Information) with anatase phase TiO_2_ (JCPDS:00‐001‐0562). The strongest diffraction peak of (220) for Ir‐CCTO NBs is noticeably shifted to a lower diffraction angle compared to that of CCTO NBs (Figure 2C), indicating the substitution of Ti^4+^ (ionic radius = 0.61 Å) ions by Ir^4+^ ions (ionic radius = 0.63 Å) in the perovskite lattice.^[^ ^28^ ^]^ Further to understand the doping effect, we synthesized the sample with IrO_2_ precursor instead of IrCl_3_·*x*H_2_O (see Experimental Section in the Supporting Information) during the synthesis of Ir‐CCTO (keeping rest of the synthesis parameter fixed). The diffraction peaks of the as‐synthesized sample matched with a mixed phase of IrO_2_ (JCPDS: 04‐009‐8479), TiO_2_ (JCPDS: 01‐084‐1286), and CCTO (JCPDS: 01‐075‐2188) (Figure S2B, Supporting Information), respectively, suggesting the formation of IrO_2_‐CCTO composite. Importantly, it should be noted that the highest intense peak position of (220) was found to be unchanged for IrO_2_‐CCTO compared to that of CCTO (Figure S5, Supporting Information), whereas this diffraction peak was shifted to a lower angle for Ir‐CCTO NBs, confirming the successful substitution of Ti^4+^ by Ir^4+^ in Ir‐CCTO NBs.

High‐resolution TEM (HR‐TEM) was employed to study the structural and chemical properties of Ir‐CCTO NBs. A high‐magnification HR‐TEM image of Ir‐CCTO NBs taken at a [100] zone axis showed an interplanar distance of 5.09 nm corresponding to the d‐spacing of the (011) crystallographic plane in the CCTO perovskite structure (the lower inset in Figure 2D).^[^ ^29^ ^]^ In addition, the SAED verified a well‐defined perovskite crystal structure (the upper inset in Figure 2D). Energy dispersive X‐ray (EDX) mapping images indicated that all the elements, including Ca, Cu, Ti, and O, were homogeneously distributed over the Ir‐CCTO NBs (Figure 2E). Notably, from the EDX mapping image of Ir, it can be seen that Ir elements exist on the top surface as well as the outer walls of Ir‐CCTO NBs. Darkfield‐scanning TEM (DF‐STEM) image further supported the idea that Ir‐based nanoclusters, such as IrO*_x_* ≈1–2 nm size are mainly formed at the outer walls of Ir‐CCTO NBs (Figure 2F). An annular bright field STEM (ABF‐STEM) was used to acquire atomic‐scale structural information for the Ir‐CCTO NBs (Figure 2G). The observed atomic arrangement in Ir‐CCTO NBs provides a unit cell of a typical CCTO perovskite structure taken along the [100] orientation, where the calculated structure is well superimposed on the observed image. Importantly, the presence of oxygen vacancies in Ir‐CCTO NBs was indeed directly evidenced by the ABF‐STEM image, as marked in green circles; red circles indicate the occupied O sites. Moreover, a high angle annular dark field‐STEM (HAADF‐STEM) image was used to obtain line profiles for the atomic intensity of A‐site (i.e., Cu and Ca, red box) and B‐site (i.e., Ti, green box) in Ir‐CCTO NBs, respectively (Figure 2H). Periodic arrangements in A‐site were confirmed by the obtained line profile; however, a sudden increase in the intensity profile was found for B‐site. This can be attributed to the atomic incorporation of Ir into Ti sites, possibly leading to the formation of oxygen vacancies. The STEM‐EDX atomic‐mapping results showed well‐ordered arrangements of Ca, Cu, and Ti in the Ir‐CCTO NBs (Figure 2I). Notably, it was hard to visualize atomic signals of Ir‐substituted Ti sites due to the Ir‐based nanoclusters existing at the surfaces. In comparison, we also performed atomic‐scale HR‐TEM analysis in combination with STEM‐EDX on the pristine CCTO NBs, where neither the formation of oxygen vacancies nor the substitution of Ti sites by Ir dopants was detectable (Figures S6–S10, Supporting Information). Overall, the introduced Ir atoms were successfully doped into the perovskite CCTO lattice by substituting Ti ions and, at the same time, some of them formed the Ir‐nanoclusters at the outer wall of CCTO NBs. Importantly, the Ir‐substituted Ti sites could create a number of oxygen vacancies modulating electronic configurations near the Fermi levels of transition metals and oxygen in the perovskite lattice, certainly beneficial for boosting charge transport during electrochemical reactions. Furthermore, the Ir‐based nanoclusters at the surface of NBs will act as efficient active sites for OER under acidic conditions. Thus, these dual functions of Ir, when introduced into the electrically semiconductive CCTO, need to be considered to understand electrocatalytic reaction mechanisms in the Ir‐CCTO NBs for OER, which will be further discussed below.

**Figure 3:**
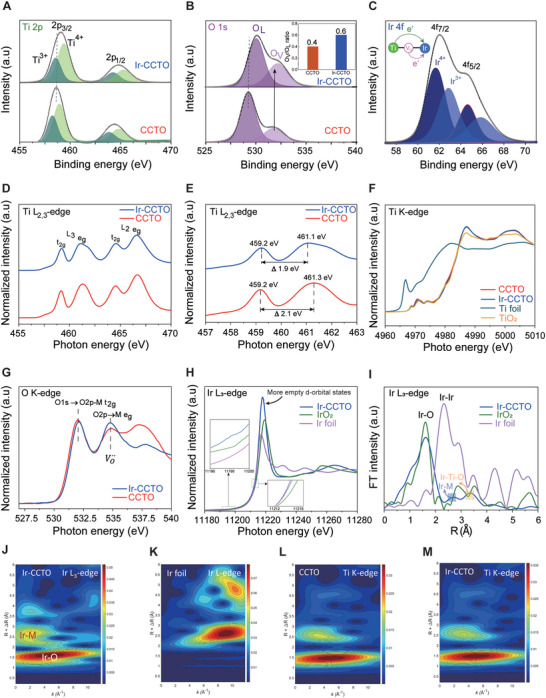
Characterization for electronic properties of Ir‐CCTO NBs. XPS spectra of A) Ti 2p, B) O1s, and C) Ir 4f for Ir‐CCTO NBs. XANES spectra of D,E) Ti L_2,3_‐edge, F) Ti K‐edge, G) O K‐edge, and H) Ir L_3_‐edge for Ir‐CCTO NBs. I) EXAFS spectra of Ir L_3_‐edge for Ir‐CCTO NBs. J,K) CCWT plots of Ir L_3_‐edge for Ir‐CCTO NBs and Ir metallic foil. L,M) CCWT plots of Ti K‐edge for CCTO and Ir‐CCTO NBs.

Modulations of the electronic configuration of CCTO after incorporation of Ir were studied by X‐ray spectroscopic analysis. First, X‐ray photoelectron spectroscopy (XPS) was performed on the CCTO NBs and Ir‐CCTO NBs. In the Ti 2p spectra of both materials, two symmetric peaks can be assigned to the 2p_3/2_ and 2p_1/2_ core levels of titanium (**Figure** **3**A).^[^ ^22^ ^]^ The spin‐orbital splitting with an energy of approximately 5.7 eV indicates the presence of Ti^4+^ species with a low valence state of Ti^3+^ as the deconvoluted peaks appeared at 458.3 and 458.6 eV for both samples (Table S1, Supporting Information). The Cu 2p spectra show the negligible difference between CCTO and Ir‐CCTO NBs, where both exhibit a mixed valence state (Figure S11, Supporting Information). As shown in Figure 3B, the O 1s XPS spectra of Ir‐CCTO NBs can be deconvoluted into two peaks that are associated with the lattice oxygen (O_L_) at 530.1 eV and the lattice sites close to the oxygen vacancy (O_V_) at 532.2 eV, respectively (Table S1, Supporting Information). Importantly, the quantitative ratio of O_V_ and O_L_ (O_V_/O_L_) peaks was substantially enhanced for Ir‐CCTO NBs (0.6) compared with that of CCTO NBs (0.4) (the inset of Figure 3B). The highly oxygen‐deficient nature of Ir‐CCTO NBs can be attributed to the structural flexibility of the pristine CCTO perovskite phase in combination with the introduction of multivalent Ir dopants.^[^ ^23^ ^]^ In addition, the O 1s spectra of Ir‐CCTO NBs show a positive shift of approximately 0.8 eV relative to that of CCTO NBs, implying stronger surface coupling after Ir incorporations. The Ir 4f spectra of Ir‐CCTO NBs display two peaks related to 4f_7/2_ and 4f_5/2_ (Figure 3C). The doublet peaks at 62.8/65.8 eV and 61.6/64.6 eV are attributed to the Ir^4+^ and Ir^3+^ species demonstrating a mixed valence state of Ir (Table S1, Supporting Information).^[^ ^30^ ^]^ Notably, the Ti 2p peaks of Ir‐CCTO NBs exhibited a slight shift toward higher binding energy than those of CCTO NBs (Figure 3A), suggesting electron transfer occurs from Ti to Ir species. In addition to the electrons from Ti, excess electrons can be also transferred from the oxygen vacancies to the neighboring Ir, resulting in electron‐deficient and ‐rich regions at the surface of Ir‐CCTO NBs, potentially advantageous for electrocatalytic reactions. Further, we performed UV–vis absorption measurements for CCTO and Ir‐CCTO NBs (Figure S12, Supporting Information) and observed a clear absorption band tail in the near‐infrared region (800–1100 nm) suggesting the increased amount of oxygen vacancies in the Ir‐CCTO NBs.^[^ ^31^ ^]^

The X‐ray absorption near‐edge structure (XANES) spectra for the Ti L‐edge were measured for CCTO NBs and Ir‐CCTO NBs (Figure 3D). Both samples exhibit L_3_ and L_2_ doublets, which are attributed to the electron transition from Ti 2p_3/2_ and Ti 2p_1/2_ states to unoccupied 3d orbitals, respectively. The L_3_ and L_2_ edges were split into t_2g_ and e_g_ peaks due to the crystal field splitting in octahedral symmetry.^[^ ^32^ ^]^ The energy gap between t_2g_ and e_g_ decreased from 2.1 eV for CCTO NBs to 1.9 eV for Ir‐CCTO NBs (Figure 3E), suggesting a charge rearrangement takes place around Ti sites as the Ir is incorporated into CCTO.^[^ ^33^ ^]^ The Ti K‐edge (Figure 3F) and Cu K‐edge (Figure S13A, Supporting Information) XANES spectra of CCTO NBs and Ir‐CCTO NBs show similar features with the TiO_2_ and CuO references, indicating the Ti^4+^ and Cu^2+^ oxidation states in the samples. The Ti K absorption edges of CCTO NBs and Ir‐CCTO NBs appeared at slightly lower energy than the TiO_2_ reference, implying the existence of Ti^3+^ species in the samples. Additionally, the absorption peak edge was positively shifted for the Ir‐CCTO NBs relative to that of CCTO NBs due to the electron transfer from Ti to Ir, as consistent with the XPS results.

The O K‐edge XANES spectra were studied to understand the electron transitions and to evaluate the metal‐oxygen (M‐O) covalency in the Ir‐CCTO NBs (Figure 3G). Two noticeable peaks were observed near 532 and 535 eV for both CCTO NBs and Ir‐CCTO NBs. The first peak is associated with the electron excitations from the O 1s core level to the unoccupied O 2p – metal t_2g_ hybrid molecular orbital, where the absorption edge at the higher photon energy indicates stronger M‐O hybridization.^[^ ^34^, ^35^ ^]^ Notably, the peak positions for the O 1s – metal t_2g_ electron transition in CCTO NBs and Ir‐CCTO NBs appeared at much higher energy than the other oxide‐based materials, including perovskites or pyrochlores.^[^ ^36^, ^37^ ^]^ Furthermore, this peak position was even more positively shifted when Ir is incorporated into the CCTO lattice, demonstrating an exceptionally high degree of M‐O hybridizations for the Ir‐CCTO NBs, inevitably related to the enhanced electrocatalytic activity.^[^ ^36^ ^]^ The second peak near 535 eV is attributed to the allowed electron transition from the O 2p to the unoccupied metal e_g_ molecular orbitals.^[^ ^36^ ^]^ This transition is strongly affected by the presence of oxygen vacancies and their concentrations.^[^ ^38^ ^]^ The peak intensity was remarkably enhanced for the Ir‐CCTO NBs compared to that of CCTO NBs, revealing the increased number of oxygen vacancies and the improved electron transition in the hybridized M‐O orbitals. These results indicate that the incorporated Ir dopants can effectively enhance the degree of covalency in the CCTO matrix with a high number of oxygen vacancies. The higher M‐O covalency generally induces the weaker localizations of electrons in transition metal oxides, thereby improving charge–transfer kinetics during electrochemical reactions.^[^ ^35^ ^]^ Moreover, considering a highly disturbed electronic configuration at the surface of Ir‐CCTO NBs, electron transfer from the adsorbed reactants to the conductive CCTO lattice would likely occur, ensuring a high catalytic activity for OER.

The Ir L_3_‐edge XANES spectra of Ir‐CCTO NBs show the white line peak position at a similar position compared to IrO_2_ but with a slight negative shift (Figure 3H and the insets), suggesting that the oxidation state of Ir in Ir‐CCTO NBs is lower than 4^+^ as consistent with the XPS results. Importantly, the white line intensity is related to the d‐orbital density of states; a higher intensity means more empty d‐orbital states.^[^ ^39^, ^40^, ^41^ ^]^ Also, it is noted that IrO*_x_* can show an increased white line intensity compared to IrO_2_, indicating the former may have more empty d‐orbital states than the latter.^[^ ^42^ ^]^ In Ir‐CCTO NBs, the surface Ir clusters seem to exist as low‐crystalline IrO*_x_*. Hence, the surface Ir in IrO*_x_* clusters can be responsible for the increased white line for Ir L_3_‐edge with more empty d‐orbital states, while the Ir doped into CCTO lattice can accept electrons from the neighboring transition metals or oxygen vacancies due to higher electronegativity, which can be referred from the XPS results. Hence, the Ir in Ir‐CCTO NBs may be able to act as an efficient acceptor for the electrons that are transferred from the neighboring transition metals or oxygen vacancies. As a result, the co‐existence of electron‐deficient TM sites and electron‐rich Ir sites is implemented at the surface of Ir‐CCTO NBs. The former will promote adsorption of the reactants and the subsequent charge transfers from the electrolyte to the bulk of Ir‐CCTO NBs, while the latter will exceed the dissociation of water molecules, providing sufficient activated reactants for OER. All these effects will secure three critical maiden factors for efficient electrocatalysis—facile adsorption, charge transfer, and abundant reactants—highlighting the potential of Ir‐CCTO NBs as an efficient electrocatalyst.

Furthermore, extended X‐ray absorption fine structure (EXAFS) spectra were employed to study the atomic coordination environment of the incorporated Ir dopant in Ir‐CCTO NBs (Figure 3I). The commercial IrO_2_ and metallic Ir foil were used as benchmarks. The Ir‐CCTO NBs exhibited a prominent peak near 1.5 Å associated with the Ir‐O scattering path.^[^ ^43^, ^44^ ^]^ The fitting of the Ir‐CCTO EXAFS data was performed in a range of 1.13–3.7 Å to observe the scattering as shown in Figure S14, Supporting Information. Table S2, Supporting Information shows the obtained parameters from the EXAFS fitting. The 1^st^ highest peak is noticed at 1.995 Å which corresponds to Ir‐O scattering. The 2^nd^ peak shows two different scatterings from Ir‐Cu and Ir‐Ca at 3.238 and 3.351 Å. Additionally, the fitting results show that the 3^rd^ EXAFS peak is the result of Ir‐Ti scattering at 3.716 Å and a double scattering of Ir‐Ti/Ir‐O at 3.879 Å. This implies that the Ir‐M bonds (M = Cu, Ca, or Ti) are present in the Ir‐CCTO NBs, which is also observed in some defective perovskite materials, supporting the successful atomic incorporation of Ir dopants in the CCTO lattice.^[^ ^45^ ^]^ Additionally, the reduced intensity of the first shell signifies the existence of coordinately unsaturated iridium sites in the Ir‐CCTO NBs with a lower coordination number than in the IrO_2_.^[^ ^20^ ^]^

The atomic dispersion of Ir in the Ir‐CCTO NBs was further analyzed by continuous Cauchy wavelet transform (CCWT) for the Ir L_3_‐edge EXAFS data (Figure 3J). Notably, the CCWT plots for the Ir‐L_3_ edge more clearly revealed the co‐existence of Ir‐O and Ir‐M bonds in the Ir‐CCTO NBs. The contour signals at 2.6 Å are correlated with the Ir‐M bonding compared to the Ir foil reference (Figure 3K); the different *k*‐space positions indicate the presence of Ir‐M (i.e., Ir‐Ti) in addition to the Ir–Ir bonds in the Ir‐CCTO NBs. The Ir‐O bond is clearly detected near 1.5 Å for the Ir‐CCTO NBs, which is consistent with the IrO_2_.^[^ ^46^ ^]^ Moreover, CCWT plots for Ti K‐ and Cu K‐edges of the Ir‐CCTO NBs did not show significant changes relative to those of the CCTO NBs (Figure 3L,M and Figure S13B,C, Supporting Information), indicating the atomic positions of Ti and Cu have not been significantly altered after the Ir incorporation. These results confirmed that the Ir dopants could simultaneously induce the formation of IrO*_x_* clusters at the surface of Ir‐CCTO NBs and the substitution with the B‐site in the CCTO perovskite crystal structure, which will consequently play dual functional roles in enhancing the catalytic performance of the Ir‐CCTO NBs.

**Figure 4:**
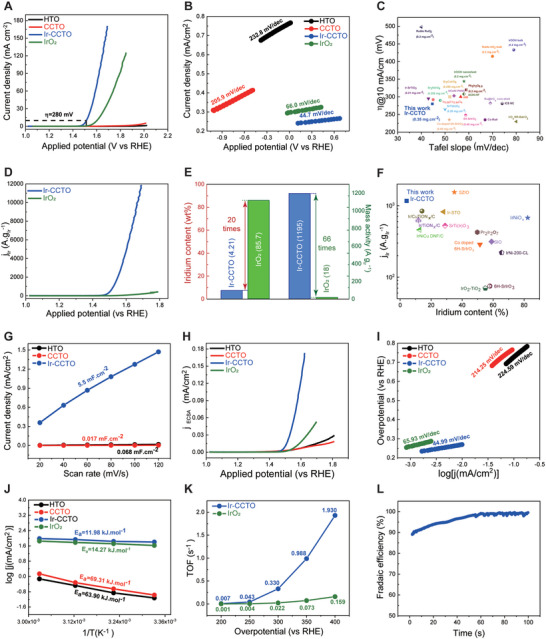
Characterization for electrochemical properties of Ir‐CCTO NBs. A) LSV polarization curves measured in 0.1 M HClO_4_ using a scan rate of 5 mV s^−1^ and B) the corresponding Tafel plots. C) Comparison of apparent catalytic activity of Ir‐CCTO NBs with other recently reported catalysts. D) Normalized currents by Ir‐mass in Ir‐CCTO NBs and IrO_2_ at different potentials. E) Ir‐mass activity of Ir‐CCTO NBs and IrO_2_ for the acidic OER at an overpotential of 300 mV. F) Comparison of Ir‐mass activity of Ir‐CCTO NBs with various recently reported catalysts for the acidic OER. G) Calculation of *C*_dl_ of Ir‐CCTO NBs and control samples. H) ECSA normalized LSV curves for the acidic OER and I) the corresponding Tafel plots. J) Activation energy for the acidic OER at an overpotential of 400 mV. K) TOFs of Ir‐CCTO NBs in comparison with IrO_2_ at different overpotentials. L) Faradaic efficient measurement of Ir‐CCTO NBs for the acidic OER using RRDE.

Electrocatalytic properties for acidic OER of the Ir‐CCTO NBs were investigated in 0.1 M HClO_4_ electrolyte. For comparison, the electrocatalytic properties of HTO, CCTO, IrO_2_, IrO_2_‐CCTO, IrO_2_‐TiO_2_, and Ir‐TiO_2_ are also studied under identical conditions. Noted that IrO_2_‐TiO_2_ and Ir‐TiO_2_ are also prepared as control samples (see Supporting Information for synthesis details and phase information in Figures S2C and S15, Supporting Information). Linear scan voltammetry (LSV) polarization curves demonstrate that the Ir‐CCTO NBs exhibit outstanding electrocatalytic activity for OER, as evidenced by the large geometric current density at small overpotentials (**Figure** **4**A). The catalytic activity of Ir‐CCTO NBs for OER was obviously superior to that of the IrO_2_ benchmark; for instance, Ir‐CCTO NBs generate a current density of 70 mA cm_geo_^−2^ at an overpotential of 370 mV, which is 7.4 times higher than that of IrO_2_ (9.5 mA cm_geo_^−2^). Moreover, two starting materials (HTO and CCTO), Ir‐CCTO NBs, IrO_2_‐CCTO, and IrO_2_‐TiO_2_ composites show negligible catalytic activity for acidic OER (Figure S16, Supporting Information). Further, Ir‐TiO_2_ shows higher electrocatalytic activity for OER than benchmark IrO_2_, however 1.2 times lower activity than Ir‐CCTO NBs. Hence, the enhanced OER activity (higher current density and lower overpotential) of Ir‐CCTO NBs can be ascribed to the synergetic effects of the incorporation of Ir dopants and the formation of surface IrO*_x_* nanoclusters in CCTO. The Ir‐CCTO NBs also have a lower Tafel slope of 44.7 mV dec^−1^ during OER compared to their counterparts including IrO_2_ whose corresponding value is 66.0 mV dec^−1^ (Figure 4B). The Tafel slope difference between Ir‐CCTO NBs and IrO_2_ may indicate the change of rate‐determining step (RDS) for OER. Specifically, the lower Tafel slope of Ir‐CCTO NBs (44.7 mV dec^−1^) infers that the RDS is related to a reaction step after the first reaction step, while the first reaction step would be the RDS for IrO_2_ as indicated by its higher Tafel slope (66.0 mV dec^−1^).^[^ ^47^ ^]^ Furthermore, based on the required overpotential (*η*) at 10 mA cm_geo_^−2^, the apparent catalytic activity of Ir‐CCTO NBs is superior or comparable to the recently reported most active Ir‐based catalysts for acidic OER (Figure 4C and Table S3, Supporting Information). Most importantly, the iridium mass activity was calculated for the Ir‐CCTO NBs and IrO_2_ using the Ir content measured from inductively coupled plasma‐optical emission spectroscopy (ICP‐OES, Table S4, Supporting Information). Notably, as shown in Figure 4D, the Ir‐CCTO NBs show much higher iridium mass activity than that of the IrO_2_. For instance, 1195 A g_Ir_^−1^ was achieved for the Ir‐CCTO NBs at an overpotential of 300 mV, which is 66 times higher than that of the IrO_2_ (18 A g_Ir_^−1^) (Figure 4E). Surprisingly, while most of the existing catalysts exhibit an iridium mass activity below 500 A g_Ir_^−1^ (Figure 4F and Table S5, Supporting Information),^[^ ^22^, ^46^, ^48^, ^49^, ^50^, ^51^, ^52^, ^53^, ^54^ ^]^ only a few catalysts, including Ir‐CCTO NBs, give an iridium mass activity higher than 1000 A g_Ir_^−1^. Overall, Ir‐CCTO NBs are one of the most active Ir‐based catalysts with ultrahigh mass activity for acidic OER. The extremely low iridium content in Ir‐CCTO NBs (4.21 wt%) further highlights its potential use in practical PEM electrolyzers.

To evaluate the intrinsic catalytic activity of iridium sites, electrochemically active surface area (ECSA) was calculated for Ir‐CCTO NBs and other control samples by measuring electrical double layer capacitance (*C*_dl_, Figure 4G and Figure S17, Supporting Information, see Experimental Section in the Supporting Information). The obtained *C*_dl_ follow the trend of Ir‐CCTO NBs (5.5 mF cm^−2^) > CCTO (0.017 mF cm^−2^) > HTO (0.068 mF cm^−2^), suggesting that Ir doping is indispensable for attaining a high number of active sites for OER. Furthermore, LSV polarization curves where the measured currents were normalized with respect to the ECSA. Importantly, the result demonstrates an exceptionally higher specific activity of Ir‐CCTO NBs for acidic OER compared to those of control samples, including the IrO_2_ benchmark (Figure 4H), highlighting the excellent intrinsic catalytic activity of Ir‐CCTO NBs. The Tafel slopes obtained by using the ECSA normalized currents decrease in the order of HTO (224 mV dec^−1^) < CCTO (214 mV dec^−1^) < IrO_2_ (65 mV dec^−1^) < Ir‐CCTO NBs (44 mV dec^−1^) (Figure 4I). These apparently evidence that the incorporation of Ir can effectively transform catalytically inert CCTO into one of the most active Ir‐based catalysts for OER under acidic conditions. The intrinsic activity of Ir‐CCTO NBs is further examined by measuring activation energy (*E*_a_, Figure 4J and Figure S18, Supporting Information), turnover frequency (TOF, Figure 4K and Figure S19, Supporting Information), and Faradaic efficiency (FE, Figure 4L and Figure S20, Supporting Information), in which all the results confirm the excellent intrinsic catalytic activity, that is, smaller activation energy, higher TOF, and nearly 100% FE, for acidic OER. The high apparent and specific activity of Ir‐CCTO NBs for OER can be attributed to the formation of active IrO*_x_* nanoclusters on the surface of NBs and the disturbed local electronic configurations at Ir‐V_O_‐M sites in the CCTO perovskite crystals. In addition, its intriguing 1D structure may offer Ir‐CCTO NBs large numbers of catalytic active sites exposed to the reactants, accounting for the high performance of OER. This can be also supported by the LSV polarization curves measured for the additional control samples, such as Ir‐CCTO nanoparticles, Ir‐CuTiO_3_, and Ir‐CaTiO_3_ (Figure S21, Supporting Information).

**Figure 5:**
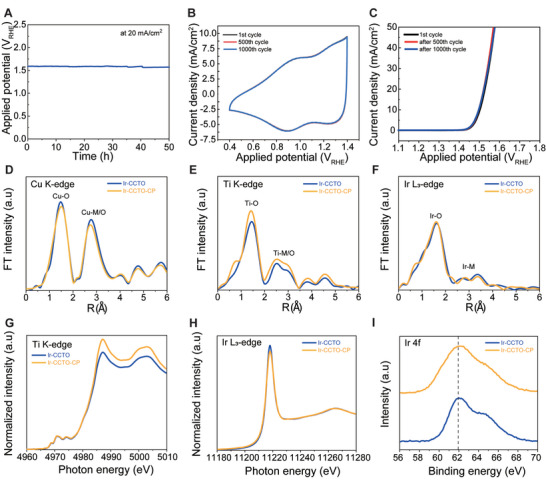
Durability test and after OER characterizations. A) CP measurement of Ir‐CCTO NBs at a constant current density of 20 mA cm^−2^ for 50 h in 0.1 M HClO_4_. B) CV cycling test of Ir‐CCTO NBs at an OER potential window up to 1000 cycling in 0.1 M HClO_4_ .C) LSV polarization curves of Ir‐CCTO NBs for the acidic OER before and after 1000 CV cycling tests. EXAFS spectra of D) Cu K‐edge, E) Ti K‐edge, and F) Ir L3‐edge for Ir‐CCTO NBs before and after CP tests. XANES spectra of G) Ti K‐edge and H) Ir L_3_‐edge for Ir‐CCTO NBs before and after CP tests. I) XPS spectra of Ir 4f for Ir‐CCTO NBs before and after CP tests.

Besides high catalytic activity, the Ir‐CCTO NBs demonstrate excellent catalytic durability for acidic OER. In chronopotentiometry (CP) measurement, an applied potential was maintained without noticeable potential increase during continuous 50 h electrolysis at 20 mA cm^−2^ under acidic conditions (**Figure** **5**A). Further, dissolved amounts of Cu, Ca, Ti, and Ir in the electrolyte during CP were quantified by ICP‐OES for the Ir‐CCTO NBs (Figure S22, Supporting Information). Electrolytes were collected during 12 h continuous CP measurement at a current density of 20 mA cm^−2^. The result showed that the leached concentration of Ir is about 0.235 ppm after 1 h electrolysis. Ca was more leached out from the Ir‐CCTO NBs, where the concentration was about 57.9 ppm. Noted that Cu and Ti were not detected as they existed below the detection limit. Importantly, the concentrations for Ir and Ca remained almost constant up to 12 h electrolysis, indicating the leaching process occurred only at the beginning of electrolysis, and the Ir‐CCTO NBs remained chemically persistent during long‐term OER in acidic conditions. In addition, the Ir‐CCTO NBs exhibited negligible changes in current density after 1000 cycles of cyclic voltammetry (CV) scans within an OER potential window (Figure 5B,C). Further, Table S6, Supporting Information demonstrates the comparison of durability decay of Ir‐based catalysts for acidic OER. The ex situ X‐ray spectroscopy and electron microscopy were performed to monitor any structural or electronic modulations in the Ir‐CCTO NBs after OER, denoted as the Ir‐CCTO‐CP. First, EXAFS and CCWT plots for Cu K‐edge, Ti K‐edge, and Ir L_3_‐edge in the Ir‐CCTO NBs and Ir‐CCTO‐CP were found to be almost similar, indicating there were no significant changes in local atomic structures of transition metals (Figure 5D–F and Figure S23, Supporting Information). The XANES spectrum of the Ir‐CCTO‐CP for transition metals also showed comparable absorption features with those of the fresh Ir‐CCTO NBs (Figure 5G,H and Figure S24, Supporting Information), demonstrating the robust electronic nature of Ir‐CCTO NBs during acidic OER. This was further supported by the XPS results for Ir‐CCTO‐CP (Figure 5I and Figure S25, and Table S1, Supporting Information). In addition, TEM, HR‐TEM, in combination with STEM‐EDX mapping, and XRD analysis, revealed the outstanding structural stability of the Ir‐CCTO NBs for OER under acidic conditions (Figures S26–S28, Supporting Information).

**Figure 6:**
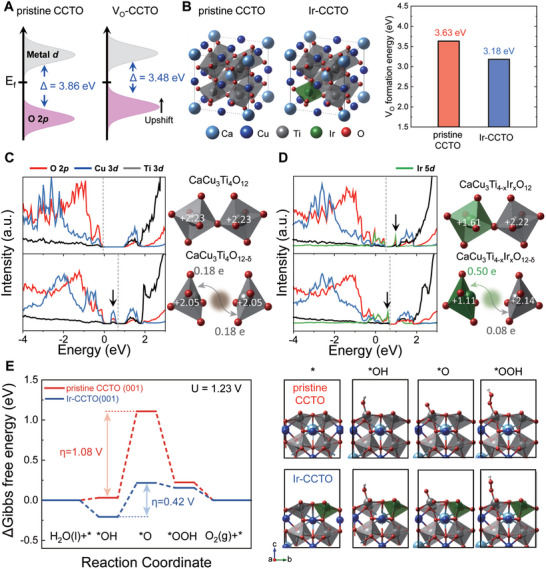
Theoretical investigations of the effect of Ir incorporation in CCTO. A) Schematic band diagrams of pristine CCTO and V_O_‐CCTO. B) Atomic configurations of pristine CCTO and Ir‐CCTO and calculated oxygen vacancy formation energies of CCTO and Ir‐CCTO. C) Projected density of states (PDOS) of O 2p, Cu 3d, and Ti 3d and Bader charge analysis for pristine CCTO and V_O_‐CCTO. D) PDOS of O 2p, Cu 3d, Ti 3d, and Ir 5d and Bader charge analysis for Ir‐CCTO and V_O_‐Ir‐CCTO. The Ir doping concentration was set to 2.5 at% (*x* = 0.5). The valence band maximum (VBM) for the pristine and stoichiometric CCTO is set to 0 eV, and the other PDOS diagrams were then aligned with respect to the core levels of the O atoms at the center of the systems. Dashed lines in PDOS diagrams indicate the VBM. E) Calculated Gibbs free energy diagram of OER pathway for pristine CCTO (001) and Ir‐CCTO (001) and their corresponding geometry‐optimized atomic configurations.

DFT calculations were also performed to better understand the role of Ir incorporation in CCTO. Detailed information about the computational methods can be found in Experimental Section in the Supporting Information. First, we theoretically measured the M‐O covalency, often referred to as the charge‐transfer energy (*Δ*), that is, the energy difference between unoccupied metal conduction bands and occupied oxygen valence bands, which has been widely utilized as a key descriptor for the OER activity^[^ ^37^, ^55^ ^]^ for the cases of pristine CCTO and CCTO with an oxygen vacancy (V_O_‐CCTO) as illustrated in **Figure** **6**A (the band structures and corresponding density of states that are used to measure the charge‐transfer energy can be found in Figure S29, Supporting Information). The calculated results show that oxygen vacancy formation significantly decreases the charge‐transfer energy down to 3.48 eV compared to the pristine CCTO, 3.86 eV, which is mainly attributed to the upshift of O 2p bands. It is well known that reduced charge transfer energy could enhance OER activity by increasing electrical conductivity and facilitating charge transfer during electrochemical reactions. Thus, we can deduce that the facile formation of oxygen vacancies in CCTO could lead to enhancing OER activity, which naturally leads to the question of how oxygen vacancy formation can be promoted.

The oxygen vacancy formation energy depending on the presence of Ir dopants in the CCTO matrix was then calculated. The atomic structures of cubic‐based ABO_3_ type perovskite oxides with 2 × 2 × 2 supercells (total 40 atoms) were used to describe the pristine CCTO and Ir‐doped CCTO (Ir‐CCTO) where the Ir doping concentration was set to 2.5 at% (Figure 6B, see Experimental Section in Supporting Information). The calculated oxygen vacancy formation energies for pristine CCTO and Ir‐CCTO were 3.63 and 3.18 eV, suggesting Ir doping facilitates the oxygen vacancy formation, which should be beneficial to the OER activity as mentioned above. We further performed Bader charge and projected density of states (PDOS) analyses to elucidate the role of Ir in promoting oxygen vacancy formation. In the case of pristine CCTO, the unoccupied Cu 3d states near CBM are responsible for the localization of excess electrons generated by oxygen vacancy formation (Figure 6C and Figure S29, Supporting Information). However, for Ir‐CCTO, the unoccupied Ir 5d states are newly formed below the unoccupied Cu 3d states and become CBM when Ir is doped in CCTO (Figure 6D and Figure S30, Supporting Information), thus promoting the oxygen vacancy formation by reducing the energy required to localize the excess electrons. The Bader charge analysis is also consistent with the PDOS interpretation showing that the larger amount of charge is transferred to Ir in Ir‐CCTO, 0.50 e (Figure 6D) compared to Ti in pristine CCTO, 0.18 e (Figure 6C) due to the electronegativity difference between Ir and Ti when the oxygen vacancy is formed. Therefore, we postulate that one of the main reasons for the enhanced OER activity of the Ir‐CCTO NBs is the increased number of oxygen vacancies promoted by Ir incorporation in CCTO leading to the reduced charge‐transfer energy, which is in good agreement with the experimental results.

In addition, the effect of Ir incorporation in CCTO on the catalytic activity toward the 4e^−^ OER pathway was investigated since Ir incorporation can also modify the surface electronic structures which essentially affects the OER activity. The Gibbs free energies of oxygen‐intermediates for the OER process were calculated for the pristine CCTO (001) surface, Ir‐doped CCTO (001) surfaces where Ir is doped at the surface and the second layer of the surface (Figure S31, Supporting Information). As shown in Figure 6E, for the pristine CCTO (001) surface, the theoretical overpotential was estimated to be 1.08 V due to the too‐weak binding of O with Ti on the surface. However, when Ir is doped at the surface, the theoretical overpotential was found to be significantly reduced to 0.42 V by enhancing O binding to Ti ions near Ir dopants, which indicates that Ir incorporation also modulates the electronic structure of the CCTO surface towards better OER activity. It was also confirmed that the Ir doping at the second layer of the CCTO (001) surface promotes the OER activity by reducing the theoretical overpotential (Figure S32, Supporting Information). We have also calculated energy diagrams for CCTO and Ir‐CCTO at *U* = 0 V (Figure S33, Supporting Information) to theoretically compare catalytic activity with other catalysts exhibiting high performance for OER under acidic conditions. Strikingly, Ir‐CCTO occupies a position on top of the OER volcano plot (Figure 1B), demonstrating catalytic activity of CCTO can be significantly provoked by adding a small amount of Ir dopants. Noted from our calculations that the substitution of Ti by Ir in a conventional cell of CCTO led to the 0.11% lattice expansion which could be difficult to be detected by the XRD technique. Further, the formation IrO*_x_* nanocluster at the surface of Ir‐CCTO NBs is indeed significant in boosting acidic OER activity. Hence, in this study, although we have identified the synergetic effects between IrO_x_ and Ir‐doping are responsible for the outstanding catalytic performance of Ir‐CCTO for the acidic OER, the quantitative analysis seems to be necessary for understanding the roles of respective phenomena.

## 3 Conclusion

In summary, the Ir‐CCTO NBs are highly active and durable electrocatalysts for the acidic OER with an ultrahigh mass activity higher than 1000 A g_Ir_^−1^ which is 66 times higher than the commercial IrO_2_ and most of the recently reported state‐of‐the‐art electrocatalysts. Both theoretical and experimental studies revealed that the Ir incorporation in CCTO not only facilitates the oxygen vacancy formation leading to the reduced charge‐transfer energy but also modulates the electronic structure of the CCTO surface towards better OER activity. Specifically, electron transfer occurs from the oxygen vacancy and Ti to the substituted Ir atoms leading to the electron‐rich Ti and electron‐deficient Ir site; the former will give rise to the favorable OER adsorption energy to the oxygen intermediates at the surface while the latter efficiently supplies electrons to the reactants. Further, the formation of IrO*_x_* nanoclusters at the surface of Ir‐CCTO NBs could significantly boost electrocatalytic activity for the acidic OER. All these effects implemented exceptionally high mass activity to the Ir‐CCTO for the acidic OER taking a top position at the OER volcano plot. This work provides a new and rational strategy for developing a high‐performance Ir‐based electrocatalyst for the acid OER using colossal dielectric materials for the first time.

## Conflict of Interest

The authors declare no conflict of interest.

## Supporting information

Supporting Information

## Data Availability

The data that support the findings of this study are available from the corresponding author upon reasonable request.
