# Supplementary material for: Colossal Dielectric Perovskites of Calcium Copper Titanate (CaCu3Ti4O12) with Low‐Iridium Dopants Enables Ultrahigh Mass Activity for the Acidic Oxygen Evolution Reaction
Source: Adv Sci (Weinh). 2023 Mar 29;10(16):2207695. doi: 10.1002/advs.202207695 (PMC10238205; doi:10.1002/advs.202207695)
Supplement: Supplementary file 1 — Supporting Information [file ADVS-10-2207695-s001.pdf]

Supplementary Materials for

Colossal dielectric perovskites of calcium copper titanate ($CaCu_3Ti_4O_{12}$) with low-iridium dopants enables ultrahigh mass activity for the acidic oxygen evolution reaction

Nguyen Thi Thu Thao[†]*, Kwangsoo Kim*[†]*, Jeong Ho Ryu*[†]*, Byeong-Seon An, Arpan Kumar Nauak, Jin Uk Jan, Kyeong-Han Na, Won-Youl Choi, Ghulam Ali, Keun Hwa Chae, Muhammad Akbar, Kyung Yoon Chung, Hyun-Seok Cho, Jong Hyeok Park, Byung-Hyun Kim*[, *] *and HyukSu Han*[, *]

Materials and Methods

Chemicals

For the synthesis of materials, Titanium(IV) oxide TiO_2 anatase (99.7%), Copper(II) nitrate hemi(pentahydrate) ($Cu(NO_3)_2.2.5H_2O$, 98%), calcium nitrate tetrahydrate ($Ca(NO_3)_2.4H_2O$, 99%), sodium hydroxide (NaOH, ACS reagent, ≥97.0%, pellets), Iridium(III) chloride hydrate ($IrCl_3.xH_2O$) were purchased from Sigma-Aldrich (Germany). Hydrochloric acid (HCl, 37%) was purchased from Deajung Chemicals & Metals (Republic of Korea). Ethanol anhydrous solution (C_2H_5OH, 99.9%) was purchased from Samchun Chemicals (Republic of Korea). All chemicals were used as purchased without further treatment.

Materials Synthesis

Synthesis of HTO nanobelts

1.88 g of TiO_2 was dispersed in 91 ml of 10 M NaOH and stirred for 24h. Then the mixed solution was relocated to a Teflon-lined autoclaves which was hydrothermally reacted at 240ºC for 24h. The resulting sodium titanate nanobelts were washed several times with DIW and then soaked in a 0.2M HCl solution and stirred for 4h. During this step, ion exchange between sodium and hydrogen can be taken palace resulting in hydrogenated titanate ($H_2Ti_3O_6$, HTO) nanobelts. Finally, the resulting precipitate was obtained after washing several times with DIW and drying at 70ºC overnight.

Synthesis of $CaCu_3Ti_4O_{12}$ (CCTO) nanobelts

To begin, 0.2 g HTO NWs, 0.396 g $Cu(NO_3)_2.2.5H_2O$ and 1.17 g $Ca(NO_3)_2.4H_2O$ (99%) were dispersed in 60 ml C_2H_5OH anhydrous. The solution was then sonicated for 30 min and saturated with nitrogen. Then, this solution was heated in an oven at 155ºC for 24h. After hydrothermal process was complete, the precipitate was collected, washed several with DIW, and dried at 70ºC overnight. The powder was transferred to furnace, and calcined in air at 500°C/600ºC/700°C/800°C for 2h with heating rate of 3ºC/min. After cooling to room temperature, the as-obtained powder was treated in 0.2M HCl for 1h to remove the copper (II) oxide (CuO). Finally, the remaining precipitate was washed three times with DIW and twice times with ethanol, and dried at 70ºC overnight.

Synthesis of Ir-CCTO nanobelts

Ir-doped CCTO NWs was synthesized by hydrothermal method. With magnetic stirring, a mixed of 0.15g CCTO and 0.2/1/5/10 % of $IrCl_3.xH_2O$ was dispersed in 60 ml DIW. Then, the

solution was transferred into a Teflon and heated at 150ºC for 24h. Finally, the product was dried in an oven at 70ºC overnight after washing three times with H_2O to obtain Ir-CCTO NWs.

Synthesis of IrO_2-CCTO composite

IrO_2-CCTO nanocomposite was synthesized by hydrothermal method. With magnetic stirring, a mixed of 0.15g CCTO and 5 % of IrO_2 was dispersed in 60 ml DIW. Then, the solution was transferred into a Teflon and heated at 150ºC for 24h. Finally, the product was dried in an oven at 70ºC overnight after washing three times with H_2O to obtain IrO_2-CCTO nanocomposite.

Synthesis of IrO_2-TiO_2 composite

IrO_2-TiO_2 nanocomposite was synthesized by hydrothermal method. With magnetic stirring, a mixed of 0.15g TiO_2 and 5 % of IrO_2 was dispersed in 60 ml DIW. Then, the solution was transferred into a Teflon and heated at 150ºC for 24h. Finally, the product was dried in an oven at 70ºC overnight after washing three times with H_2O to obtain IrO_2-TiO_2 nanocomposite.

Synthesis of Ir-TiO_2 composite

The as-prepared HTO (0.2 g) and 0.037 g of $IrCl_3$ were mixed in 100 ml of DI water by stirring for 10 h followed by 20 min of sonication. The mixed solution was transferred into a Teflon-lined autoclave for a hydrothermal reaction at 200 °C for 24 h. The resulting powder was washed with DI water theree times, collected via centrifugation, and dried at 70 °C.

Characterizations

Scanning electron microscopy

SEM (model S4800; Hitachi) was performed on the samples to investigate the structural parameters.

Transmission electron microscopy

TEM (Talos F200X; Thermo Fisher Scientific) was employed to collect high-resolution TEM images with elemental distributions using an equipped energy dispersive X-ray (EDX) spectroscopy.

Atomic-scale transmission electron microscopy

Atomic-scale TEM was perforemd by Cs-corrected TEM (JEM-ARM200F NEOARM; JEOL) equipped with a cold field emission gun (CFEG) and EDX (JED-2300T; JEOL) at 200 keV. Dual-beam focused ion beam (AURIGA CrossBeam Workstation; Carl Zeiss) was employed

to acquire atomic-scale scanning TEM (STEM) images with a EDX chemical mapping information using JEOL-EDX in the STEM imaging mode, and each detector has an effective detection area of 100 mm^2. The sample drift during the acquisition was tried to eliminate by tracking the reference atom position which was determined at the starting of the measurement.

Powder X-ray diffraction

XRD data were collected with Cu Kα radiation under the operating set-up of 40 kV and 100 mA using a X-ray diffractometer (Rigaku D/Max 2550).

X-ray photoelectron spectroscopy

XPS (VG ESCALAB 200i; Themo Fisher Scientific) was employed to track the samples' surface electronic structure. The spectrometer energy calibration was calibrated using the C peak position. Pass energies of 100 eV and 20 eV were utilized for survey and high-resolution scans, respectively.

Inductively coupled plasma-optical emission spectroscopy

Ir amount in the samples were calculated via inductively coupled plasma-optical emission spectroscopy (ICP-OES; Thermo Scientific; iCAP6500 Duo). RF power and the wavelength were set as 1350 W and 214.423 nm, respectively. The samples were firstly dissolved in a mixed solution of nitric and hydrochloric acid and heated at 150°C for about 30 min. Then, hydrofluoric acid was added to fully dissolve all materials and heated at 150°C until the solution becomes transparent.

X-ray absorption spectroscopy

XAS were measured at the 1D-PAL-KIST beamline of the Pohang Accelerator Laboratory (PAL). A double crystal monochromator (DCM) Si(111) was employed at 1D beamline and measurements were taken with a ring current of 300 mA. The harmonics of the incident X-ray beam were detuned by DCM. The energy was calibrated using reference metallic foils prior to measurements. The obtained XAS data were acquired in transmission mode and the Ti, Cu, and Ir metallic foils were used as reference. The acquired XAS data were analyzed using ATHENA software.[1-2] In order to observe the wavevector and interatomic distance (R) data in three dimensions, Continuous Cauchy wavelet transform (CCWT) analysis were accomplished with k^2-weighted signals with a k-space range of 2.0 – 12.0 $Å^{-1}$. Near edge X-ray absorption fine structure (NEXAFS) spectra of O K-edge and Ti $L_{3,2}$ edge were measured at 10D beamline with bending magnet at PAL. NEXAFS measurements were taken at room

temperature with a resolution of 0.01 eV and the spectra were acquired in a total electron yield (TEY) mode under a base pressure of 3×10^{-10} Torr.

Electrochemical Measurements

Preparation of catalyst inks and electrodes

The catalyst ink was prepared through a dispersion of 5 mg of the catalyst in 750 µL of DIW and 250 µL of ethanol containing 20 µL of Nafion 117 solution. After 30 min sonication, 5 µL of the catalyst ink was coated on a clean surface of glassy carbon electrode (GCE) with diameter of 3 mm yielding a loading amount of approximately 0.35 mg cm^{-2}. The GCE was then dried at room temperature under air.

Evaluation of catalytic activity for the acidic OER

The electrocatalytic properties of the samples were measured in 0.1M $HClO_4$ electrolyte using a three-electrode cell connected to a potentiostation (model Autolab PGSTAT; Metrohm). A rotating disk electrode (RDE) was utilized to investigate the electrochemical properties of the catalysts. A typical three-electrode setup (Pt as counter and Ag/AgCl as reference electrodes) was employed for electrochemical tests. The recorded potentials were re-calculated against the reversible hydrogen electrode (RHE). Electrolyte (0.1 M $HClO_4$) was purged with Ar_2 gas for approximately 30 minutes. In addition, 50 cycles of cycling voltammetry (CV) scans were performed in the OER potential window to electrochemically stabilize the catalysts' surface. Then, linear sweep voltammetry (LSV) curves were recorded at a 5 mV s^{-1} scan rate. In addition, the electrochemical impedance spectroscopy (EIS) was performed at 1.4V_{RHE} in a frequency range from 10 0000 Hz to 0.1 Hz. All LSV polarization curves were *iR*-corrected using the solution resistance (R_s) measured by EIS. Tafel plots were derived from the *iR*-corrected LSV polarization curves where the Tafel slopes were calculated from the equation: $\eta = b \log j + a$ where (b: Tafel slope, j: current density, η: overpotential).

Determination of electrochemical double layer capacitance (C_{dl})

CVs were measured in the non-Faradic potential range (e.q., 0.83 ~ 0.93 V_{RHE}) with a different scan rates. The difference of anodic and cathodic currents ($\Delta J = J_{anodic}-J_{cathodic}$) at the middle of potential (0.88 V_{RHE}) was plotted against the scan rate in which the slope corresponds to twice the C_{dl} of the catalyst.

Determination of electrochemical surface area (ECSA)

The ECSAs of sample was obtained from the measured C_{dl}. Notably, the charging of double layer is originated from the non-Faradaic currents which has a linear relationship with the active surface area; the $1 cm^2$ of flat surface area has a specific capacitance which is equal to C_{dl} value of 40 μF cm^{-2}.[3] Therefore, the C_{dl} is directly related with the ECSA as: ECSA = C_{dl} of catalyst (mF cm^{-2})/0.04 (mF cm^{-2}).

Determination of Ir mass activity for OER

For the mass activity calculations, the current density at a certain potential in LSV polarization curves was normalized with total mass of Ir loaded on GCE electrode which was determined from the ICP-OES results.

Determination of turn over frequency (TOF)

TOF was calculated according to the equation: $\mathrm{TOF} = \mathrm{j} \times \frac{\mathrm{A}}{4 \times \mathrm{F} \times \mathrm{N_s}}$, where j is the current density at a certain potential (Acm^{-2}), A is surface area of the working electrode (cm^{-2}), F is Faraday constant 96,458 C mol^{-1} , and N_s is concentration of active sites (mol cm^{-2}).[4] The value of Ns was determined by CV measurements where the oxidative peak currents generated by Ir has a linear relationship with a different scan rate. Here, the slope in linear plot is equal to: $\mathrm{slope} = \mathrm{n^2 F^2 A N_S / 4RT}$, in which n, F,A, Ns, R, T are the number of electrons transferred, Faradic constant, the surface area of the electrode, the surface concentration of active sites, the ideal gas constant, and the temperature, respectively.

Determination of Faradaic efficiency using rotating ring disk electrode (RRDE)

First, the collection efficiency for our RRDE system was estimated in 0.05 M Na_2SO_4 dissolved with 4 mM of potassium ferricyanide, $K_3Fe(CN)_6$, electrolyte. It can be noted that the ferrocyanide/ferricyanide half reaction occurs thorugh a simple and single-electronic reaction, and thus often used as the standard mean for estimating collection efficiency of a certain RRDE equipment.[5]

$\mathrm{Fe(CN)_6^{3-} + e^- \rightarrow Fe(CN)_6^{4-}}$ (reduction of ferricyanide)

$\mathrm{Fe(CN)_6^{4-} + e^- \rightarrow Fe(CN)_6^{3-}}$ (oxidation of ferrocyanide)

Initially, CV of bare GCE of RRDE was conducted using a scan rate of 100 mV/s without rotation. Then, LSV was perfomred on the GCE by applying potentials from 0 to 1.23 V_{RHE} at a scan rate of 50 mV/s, while a constant potential of 1.20 V_{RHE} was applied on the Pt-ring disk. RRDE voltammograms were recorded at different rotation rates such as 400, 625, 900, 1225, 1600, and 2025 rpm. The collection efficiency of RRDE system can be calculated from the

ratio between the ring limiting current and the disk limiting current, $N_{collection\ efficiency}$ = -$i_{limiting,ring}/i_{limiting,\ disk}$, which was about 0.23 for our RRDE system. To calculate Faradaic efficiency (FE) for OER, a ring potential of 0.4 V_{RHE} was applied to the Pt-ring disk to reduce the O2 molecules evolved at the disk-electrode where a constant current of 1.10 mA was applied. FE can be calculated from: FE = $i_{ring}/(i_{disk}\times N)$, where i_{disk}, i_{ring}, and N are disk current, ring current, and current collection efficiency, respectively.[5]

Density Functional Theory (DFT) Calculations

Spin-polarized DFT calculations were conducted by using the Vienna ab initio simulation package (VASP) [6-9]. The projector augmented wave (PAW) method and exchange-correlation functional of the generalized gradient approximation (GGA) followed by the work of Perdew-Burke-Ernzerhof (PBE) were used [10-12]. The H $1s^1$,O $2s^22p^4$, Ca $3s^23p^64s^2$, Ti $3s^23p^64s^23d^2$, Cu $3p^64s^13d^{10}$, Ir $5d^76s^2$ electrons were explictly treated as valence states. The energy cutoff of 500 eV was adopted. The criteria of electronic energy convergence and geometry optimization convergence were 10^{-6} eV and 0.01 eV·$Å^{-1}$, respectively. The Hubbard correction scheme [13] was applied to explicitly correct the Cu 3d and Ir 5d states with U_{eff} of 4 eV and 2 eV, respectively, taken from references [14,15]. The atomic structures of cubic-based ABO_3 type perovskite oxides with 2 × 2 × 2 supercells were used to simulate the bulk $CaCu_3Ti_4O_{12}$ system. The Monkhorst-Pack scheme with 5 × 5 × 5 *k*-points meshes was adopted to sample the Brillouin zone for bulk systems [16]. The computational methods used in this work well-reproduced the lattice constant and the magnetic moment on CuO_4 in CCTO compared with previous studies (Table S7). The oxygen vacancy formation energy, $E_{for}(V_O)$ was calculated from Equation (1):

$$E_{for}(V_O, q) = E_{tot}(V_O) - E_{tot}(perfect) + N_{V_O}\mu_O \quad (1)$$

where E_{tot} indicates the total energy of an oxygen-deficient or perfect system. N_{V_O} and μ_O represent the number of oxygen vacancies and the chemical potential of oxygen, respectively.

To calculate the adsorption free energy (ΔG_{ads}) of oxygen-intermediates for the OER pathway, (001) surface which is the most stable surface for cubic-based ABO_3 perovskite oxides was chosen [17-20]. The slabs were simulated with seven layers. The bottommost two layers were fixed maintaining the optimal bulk structures, whereas the top five layers were fully relaxed.

A large vacuum region of 20 Å in the z-direction perpendicular to the surface was constructed to avoid interactions between neighboring supercells. The k-point sampling of 5 × 5 × 1 grids was used for the surface models. The exposed B site metal, Ti was selected as an active site for the OER [21,22]. In an acidic solution, the OER is followed as a four-step process:

$$* + H_2O\ (l) \rightarrow * OH + H^+ + e^- \quad (2)$$

$$* OH \rightarrow * O + H^+ + e^- \quad (3)$$

$$* O + H_2O(l) \rightarrow * OOH + H^+ + e^- \quad (4)$$

$$* OOH \rightarrow * + O_2(g) + H^+ + e^- \quad (5)$$

where * denotes an active site on the substrate. Adsorption free energy (ΔG_{ads}) was calculated from Equation (6):

$$\Delta G_{ads} = E_{tot}(sub + ads) - \{E_{tot}(sub) + E_{tot}(ads)\} + \Delta E_{ZPE} - T\Delta S - neU + \Delta G_{sol} \quad (6)$$

where $E_{tot}(sub + ads)$ is the total energy of substrate with an adsorbate. $E_{tot}(sub)$ and $E_{tot}(ads)$ indicate the total energy of the substrate and adsorbate in a vacuum, respectively. ΔE_{ZPE}, T, and ΔS represent the difference in zero-point energy between the adsorbed state and the gas phase, temperature and change of entropy, respectively, and these values for gas-phase molecules at 300 K were directly taken from thermodynamic tables. The zero-point energies for adsorbants were obtained by vibrational frequencies calculations. n, e, and U denote the number of electron transferred, electric charge, and applied potentail, respectively. ΔG_{sol} is the term of solvation corrections taken from previous literature [23,24]. The theoretical overpotential (η) was calculated from Equation (7):

$$\eta = \max[\Delta G_i]/e - 1.23\ \mathrm{V} \quad (7)$$

where ΔG_i (i = 1 ~ 4) is the free energies of four steps (Equation (2)-(5)), and 1.23 V is the equilibrium electrode potential at pH = 0.

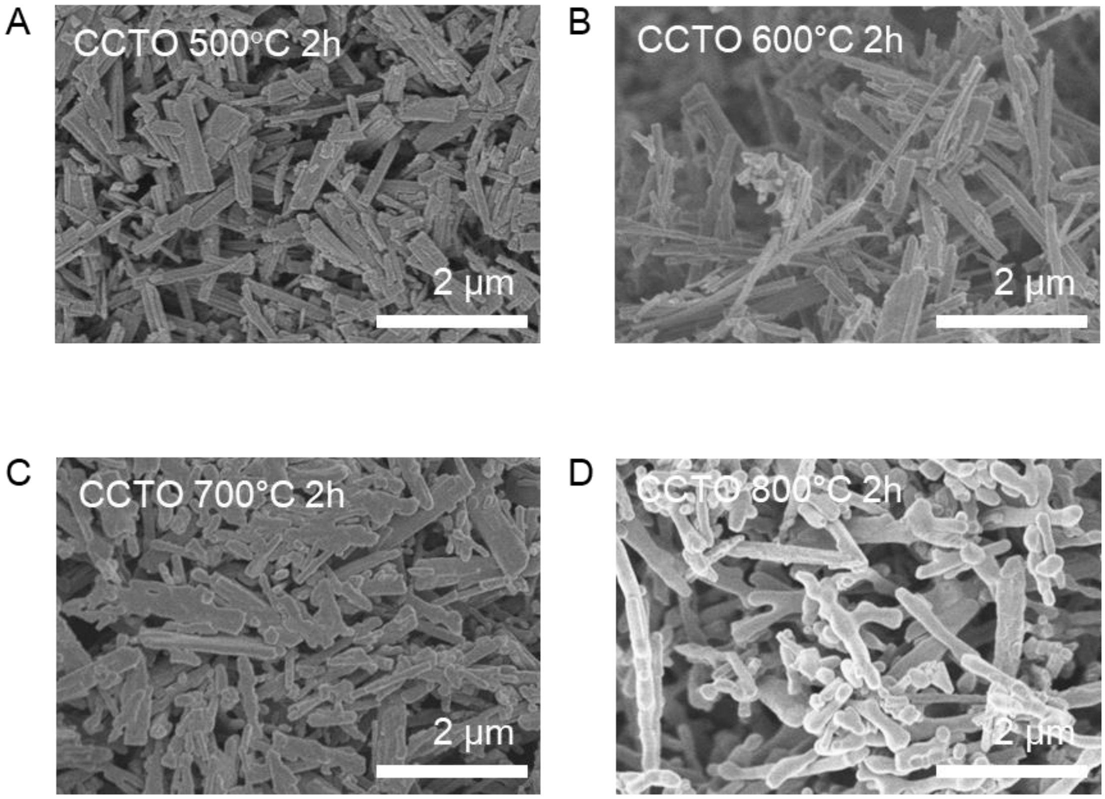

Fig. S1. SEM images of CCTO nanobelts synthesized at different temperature.

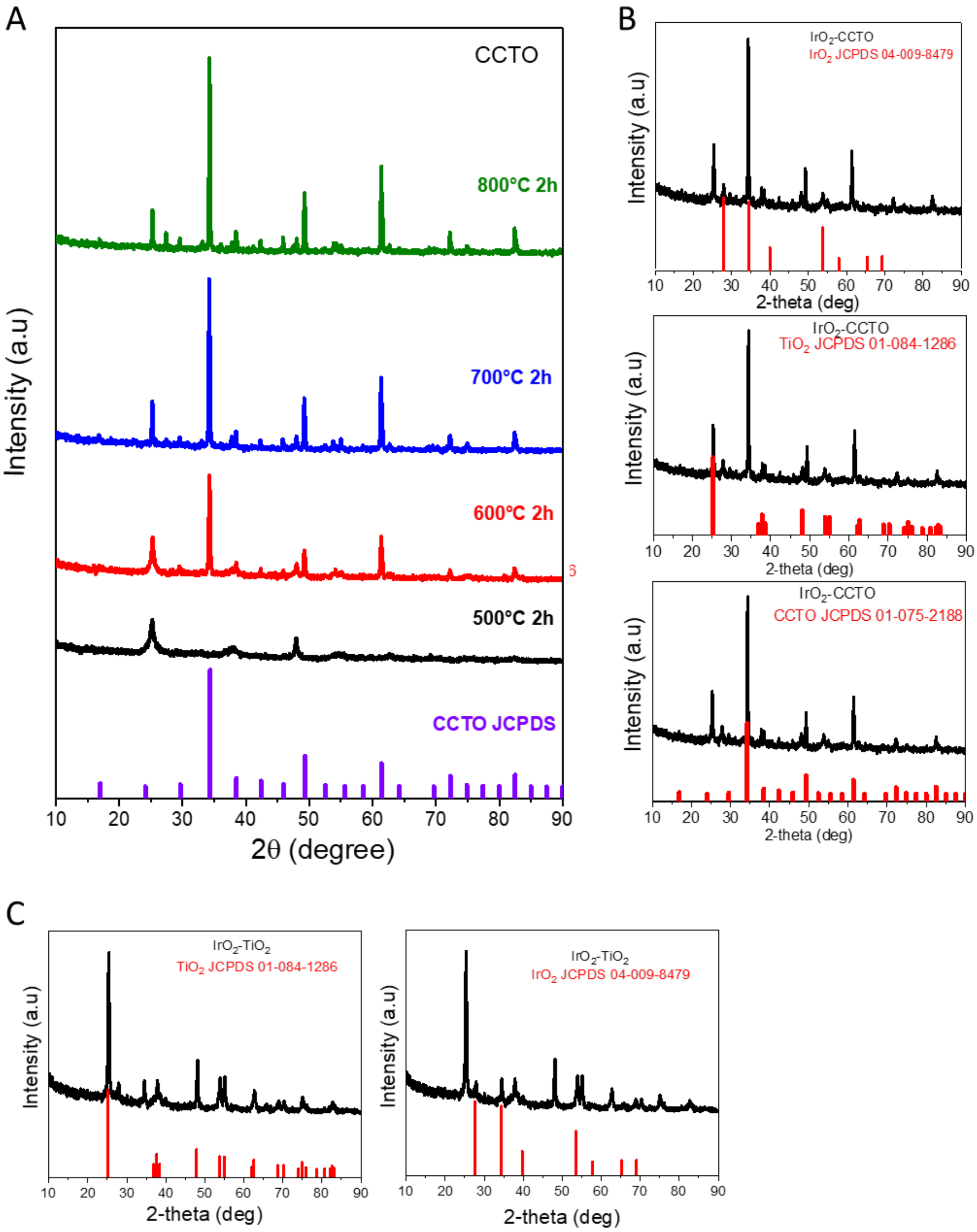

Fig. S2. Powder XRD patterns of (**A**) CCTO nanobelts synthesized at diffrenet temperature, (**B**) IrO_2-CCTO composite, (**C**) IrO_2-TiO_2 composite.

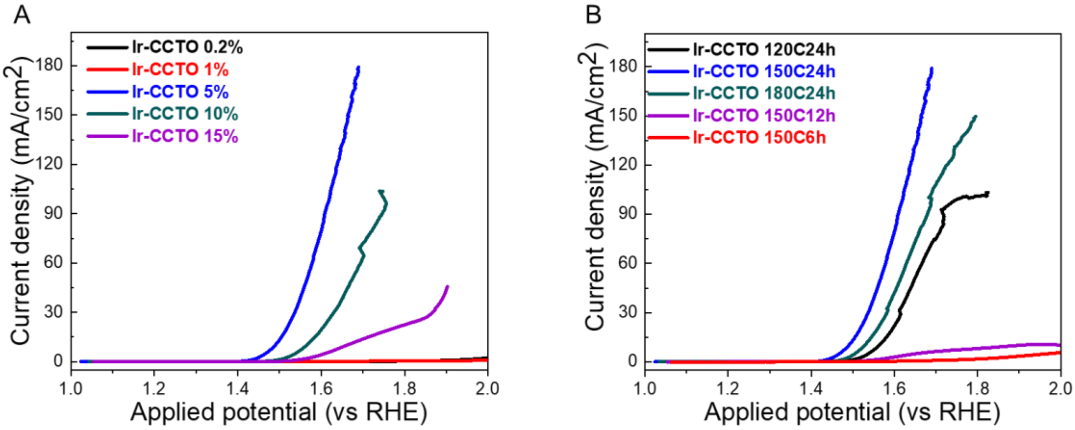

Fig. S3. LSV poralization curves of Ir-CCTO nanobelts with different Ir-content for the acidic OER.

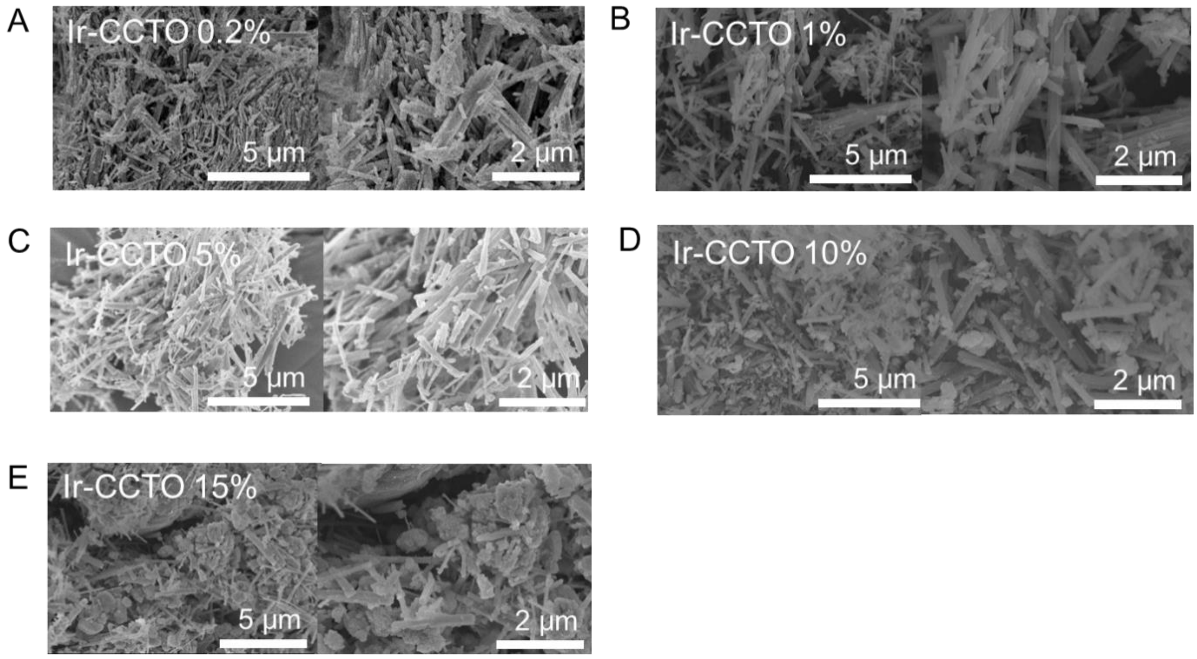

Fig. S4. SEM images of Ir-CCTO nanobelts with diefferent Ir-content.

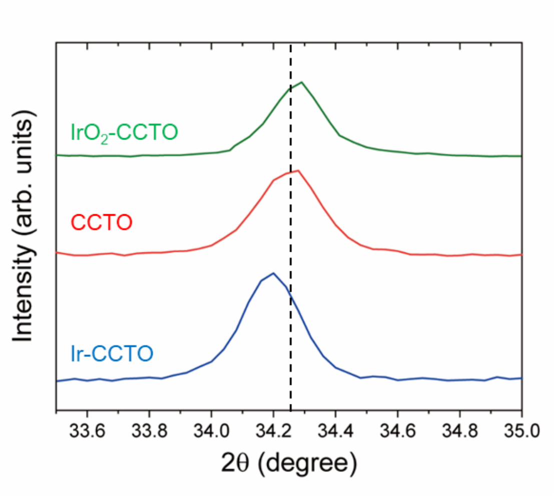

Fig. S5. Enlarged (220) diffraction peak for CCTO, Ir-CCTO, and IrO_2-CCTO composite.

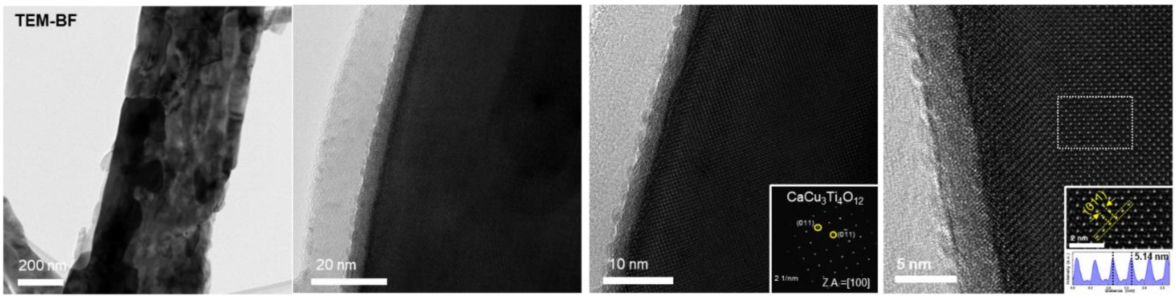

Fig. S6. TEM and HR-TEM images of CCTO nanobelts.

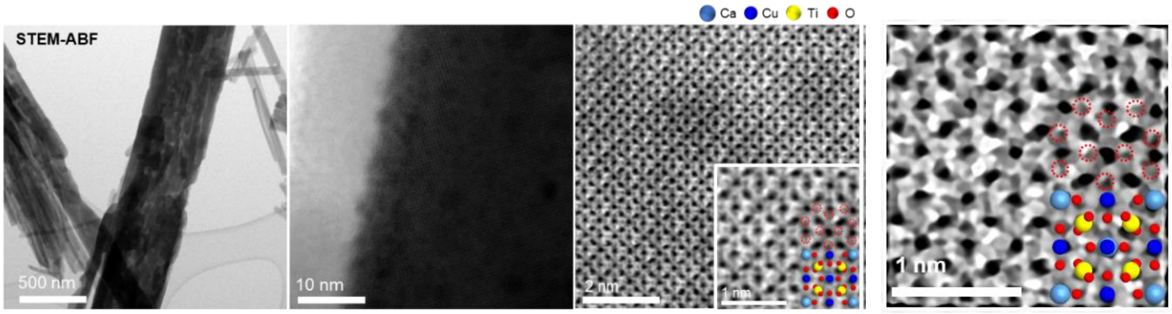

Fig. S7. Annular bright field-STEM analysis for CCTO nanobelts.

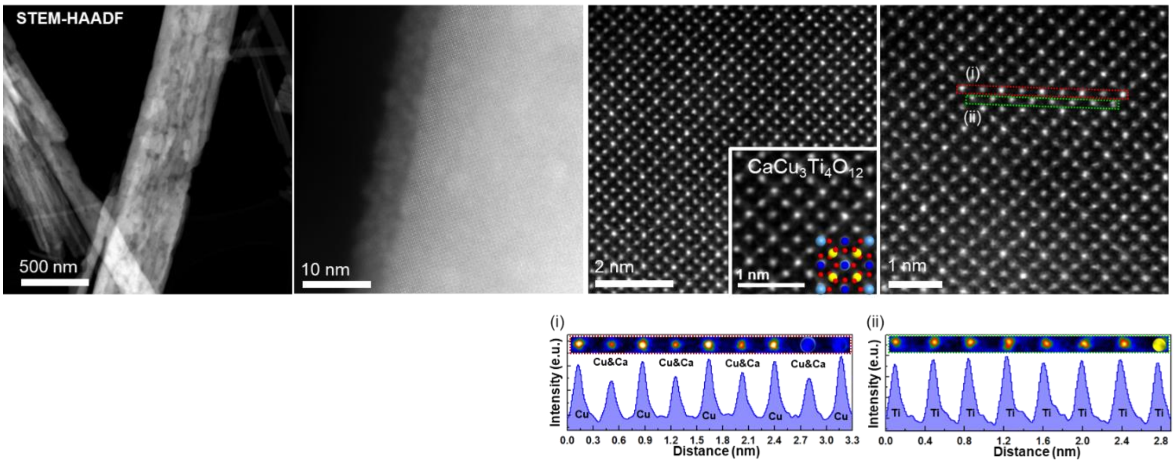

Fig. S8. High angle annular dark field-STEM analysis for CCTO nanobelts.

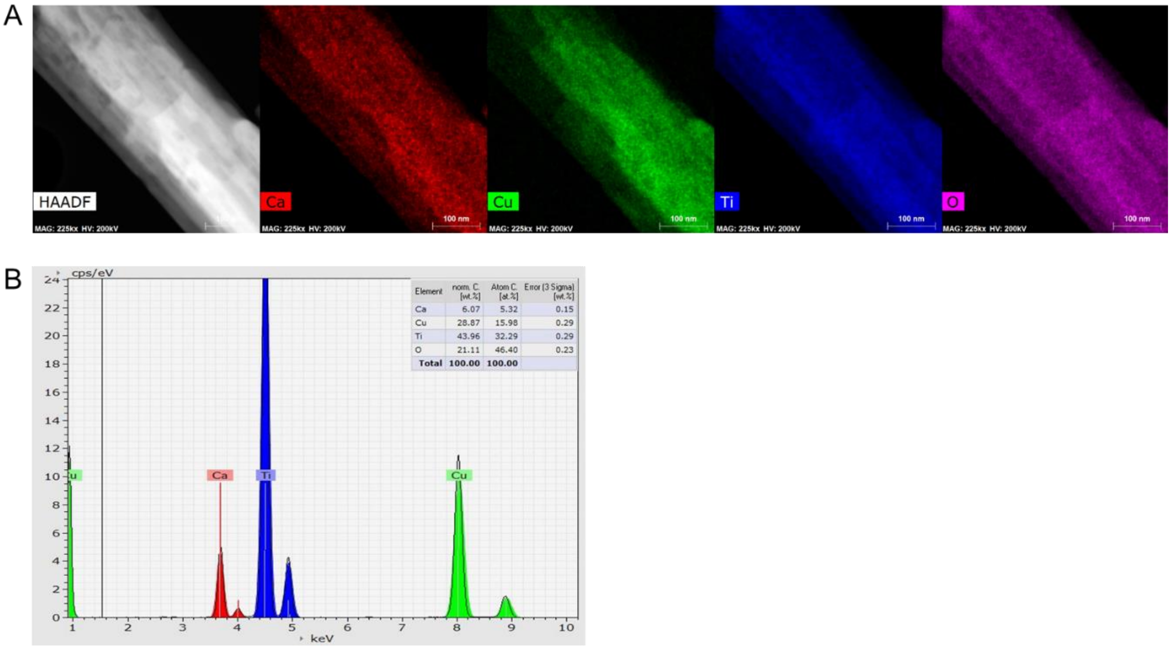

Fig. S9. (**A**) STEM-EDX elemental mapping of CCTO nanobelts and (**B**) the corresponding quantitative spectrum.

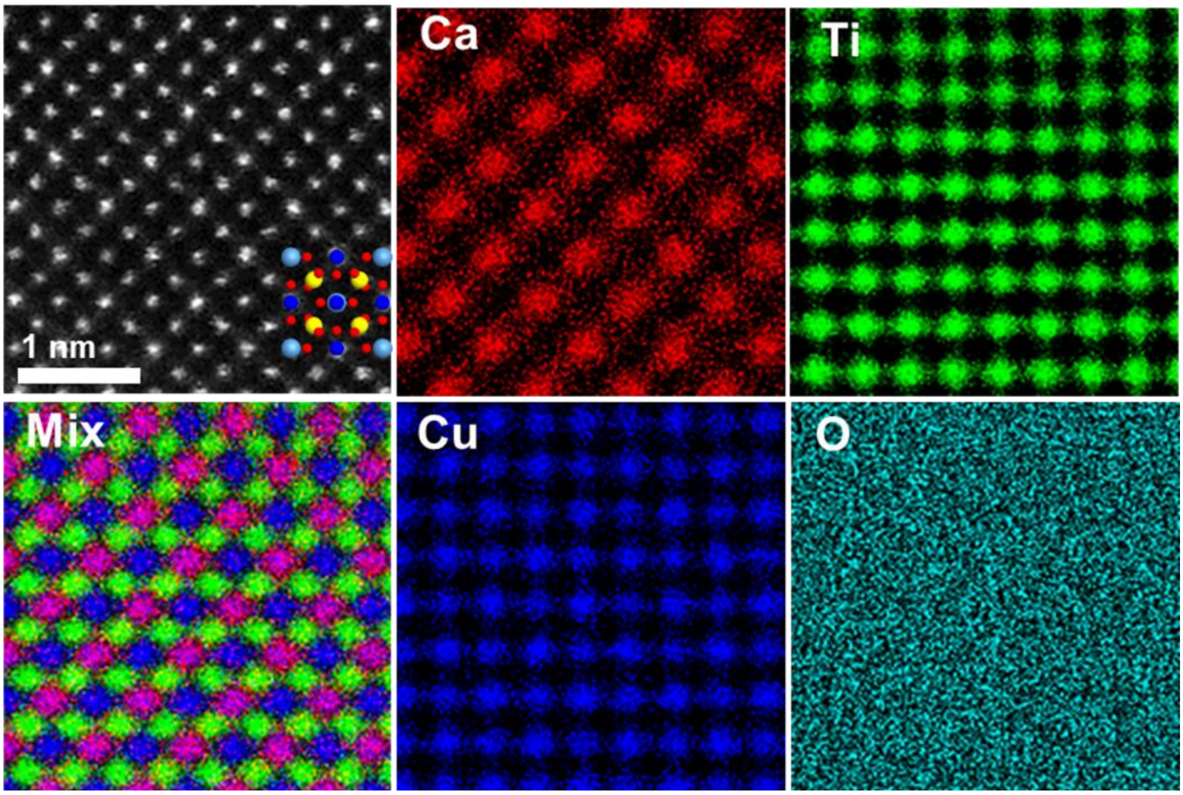

Fig. S10. Atomic-scale STEM-EDX mapping images for Ca, Ti, Cu, and O in CCTO nanobelts.

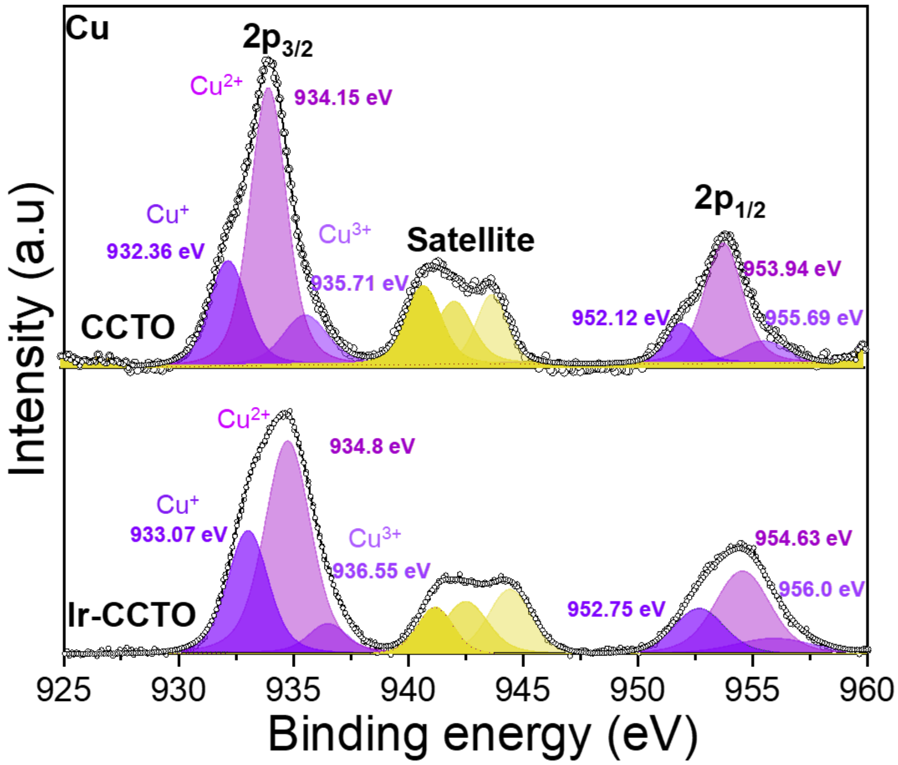

Fig. S11. XPS spectra of Cu 2p for CCTO and Ir-CCTO nanobelts.

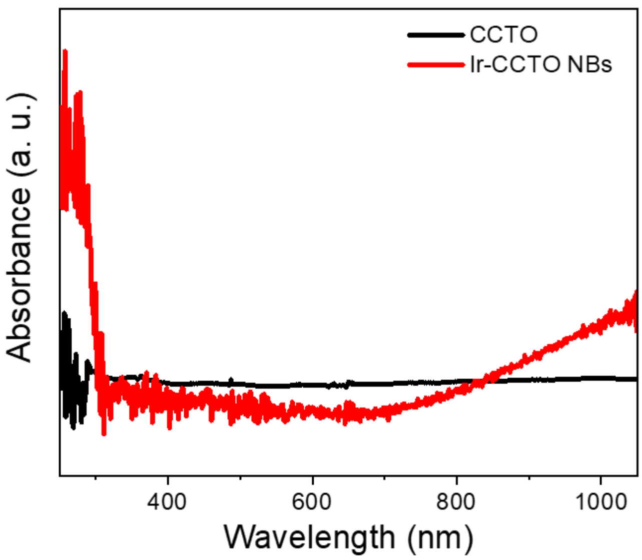

Fig. S12. UV visible spactra of CCTO and Ir-CCTO.

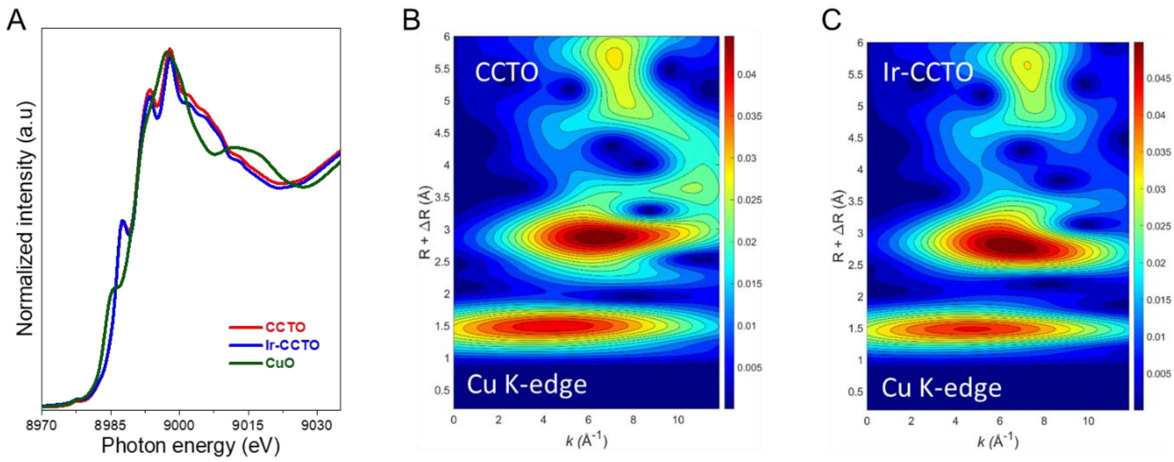

Fig. S13. (**A**) XANES spectra of Cu K-edge for CCTO and Ir-CCTO. CCWT plots of Cu K-edge for (**B**) CCTO and (**C**) Ir-CCTO.

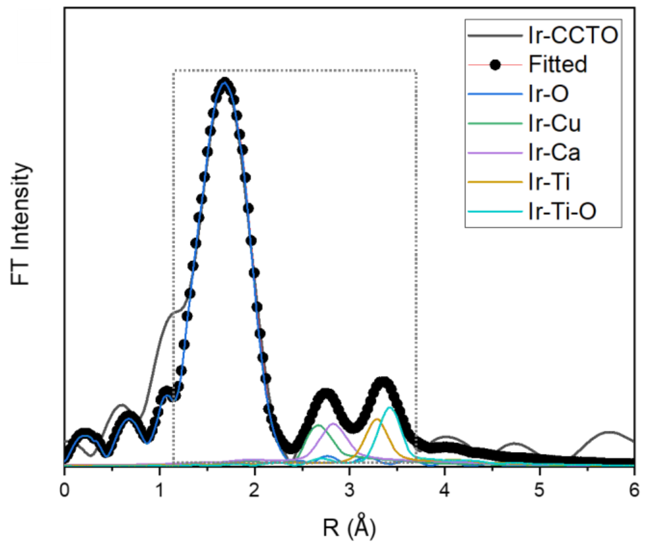

Fig. S14. Fitted EXAFS data for Ir L_3-edge of Ir-CCTO NBs.

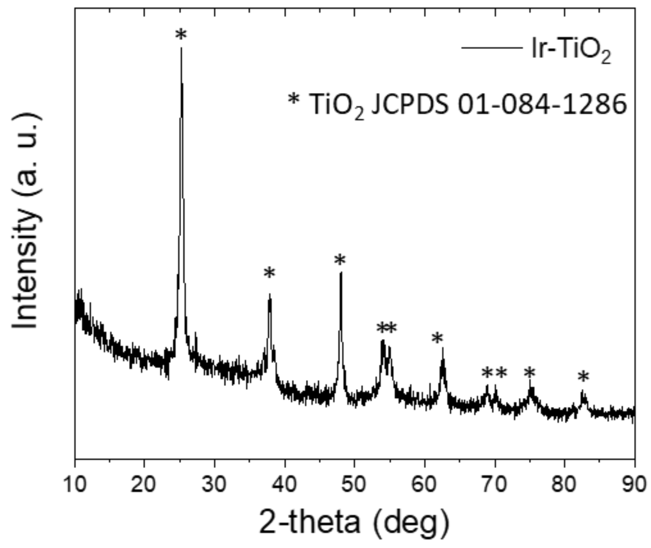

Fig. S15. XRD pattern of Ir-TiO2.

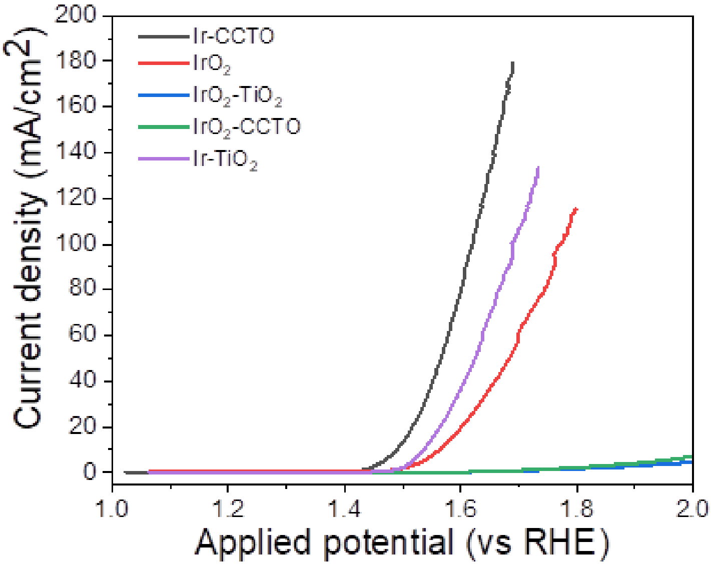

Fig. 16. LSV polarization curves measured in 0.1 M $HClO_4$ using a scan rate of 5 mV s^{-1}.

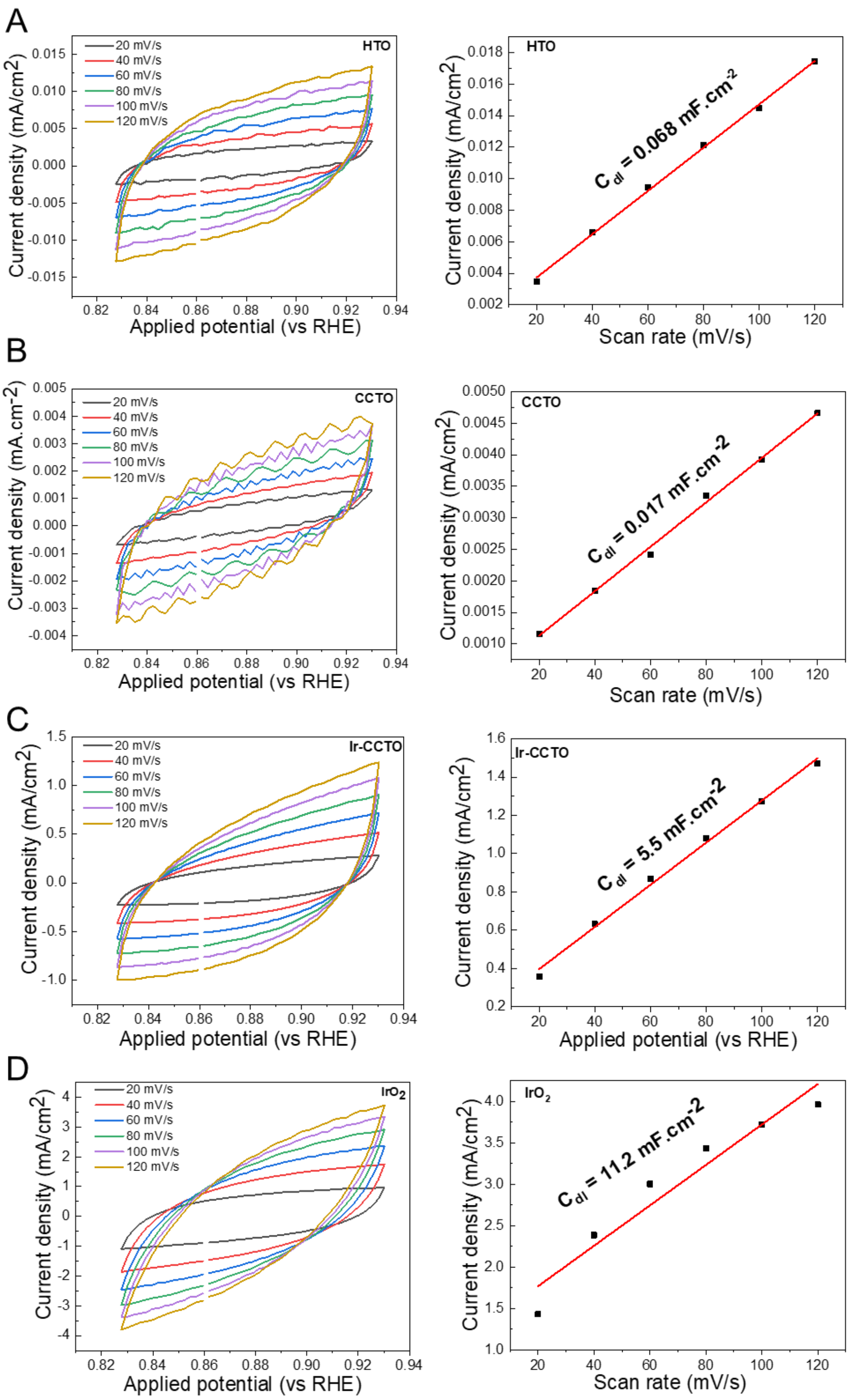

Fig. S17. Electrical double layer capacitance measurement for (**A**) HTO, (**B**) CCTO, (**C**) Ir-CCTO, and (**D**) IrO_2. (**left panels**) CV scans at different scan rates in a potential window where capacitive currents flow. (**right panels**) Plots of current difference between anodic and cathodic sweep as a function of scan rate correspond to the values of C_{dl}.

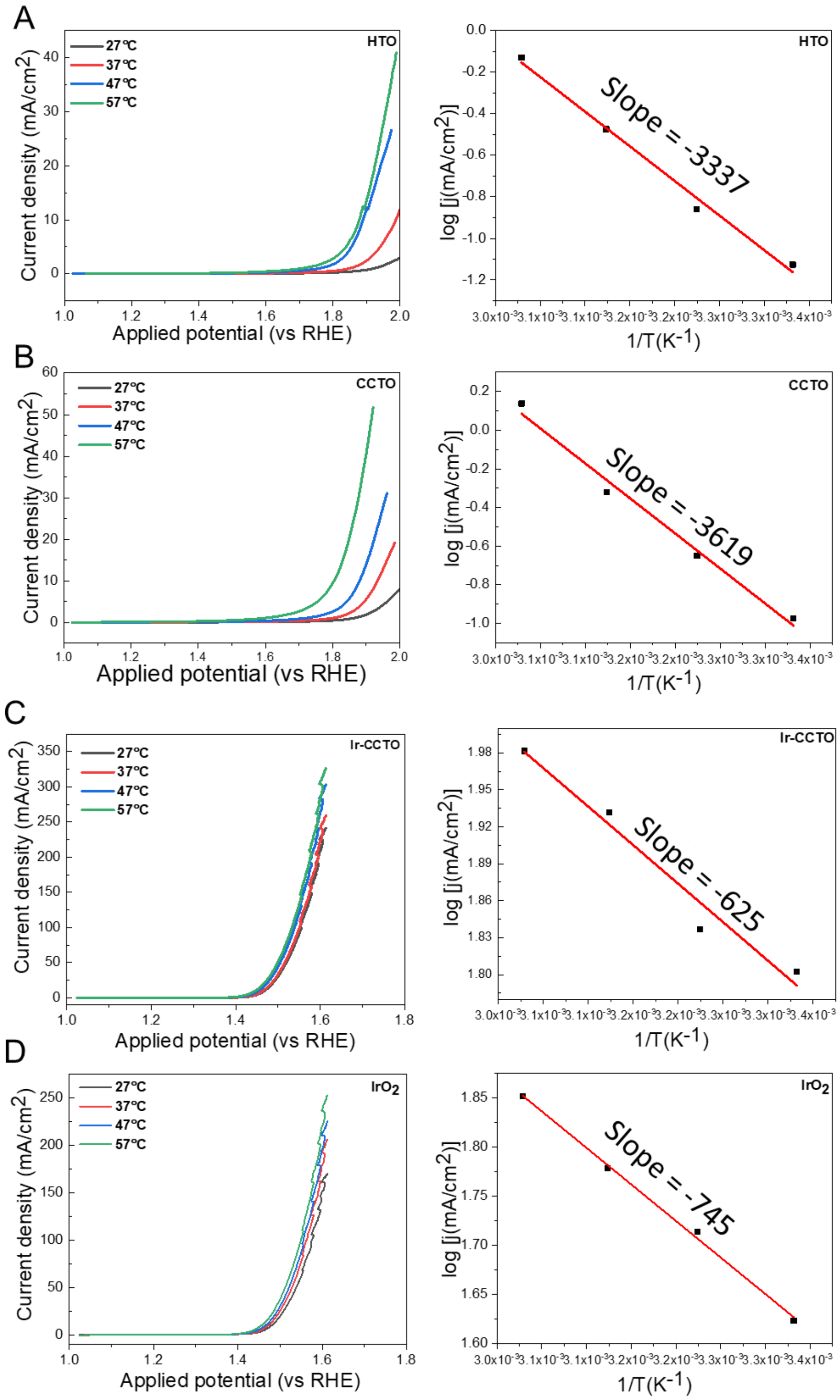

Fig. S18. Activation energy measurements for (**A**) HTO, (**B**) CCTO, (**C**) Ir-CCTO, and (**D**) IrO_2. (**left panels**) OER polarization cures measured in 0.1 M $HClO_4$ using a scan rate of 5 mV s^{-1}. (**right panels**) Arrhenius plot of current density at an overpotential of 400 mV where the slope is related with the activation energy for OER.

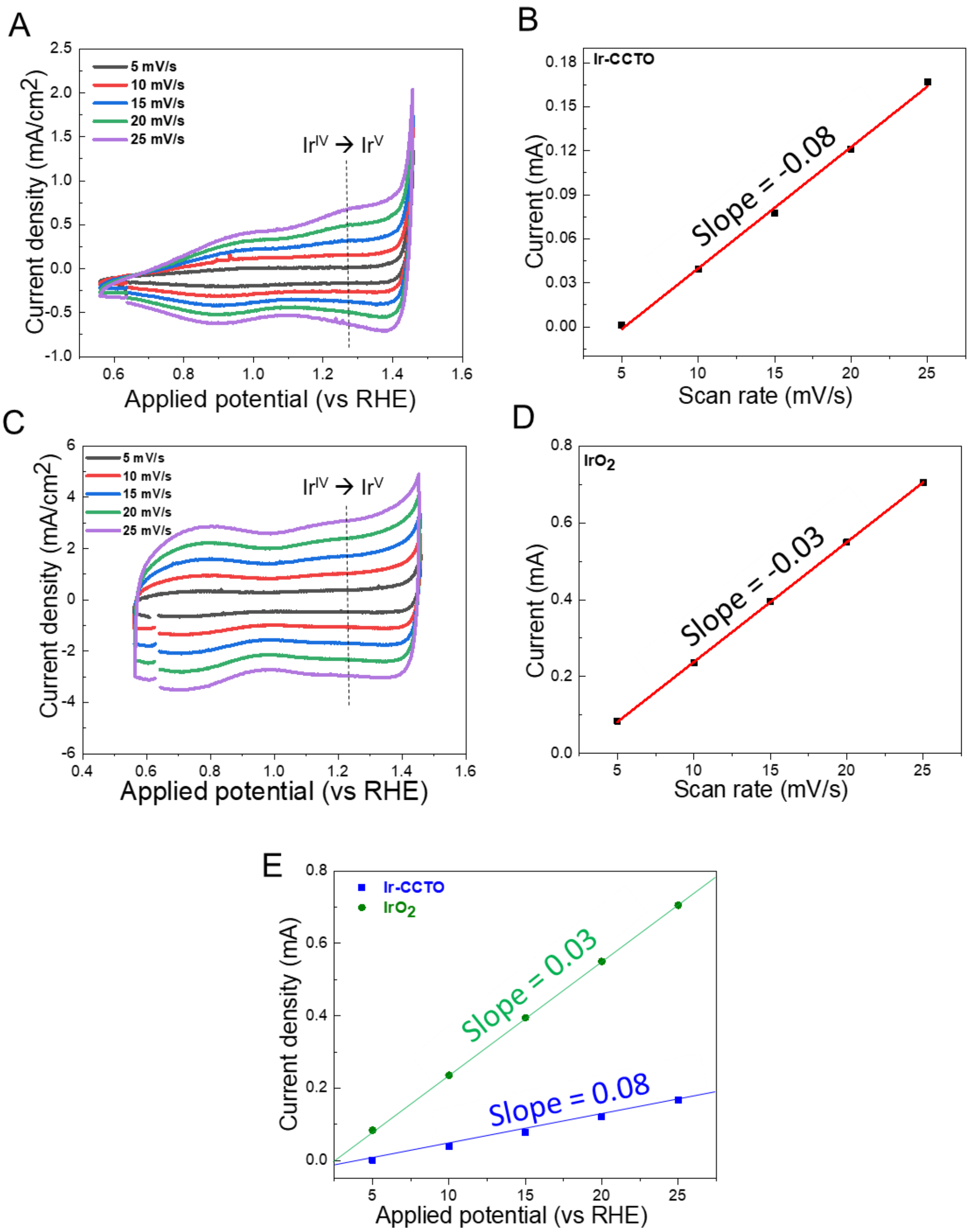

Fig. S19. TOF measurements for Ir-CCTO and IrO_2. (**A-B**) CV scans of Ir-CCTO at different scan rates in an OER potential window and the corresponding linear plot between current at 1.27 V_{RHE} and scan rates where the slope is related with the active Ir sites for OER. (**C-D**) CV scans of IrO_2 at different scan rates in an OER potential window and the corresponding linear plot between current at 1.24 V_{RHE} and scan rates where the slope is related with the active Ir sites for OER. (**E**) Comparison of the linear plots for Ir-CCTO and IrO_2.

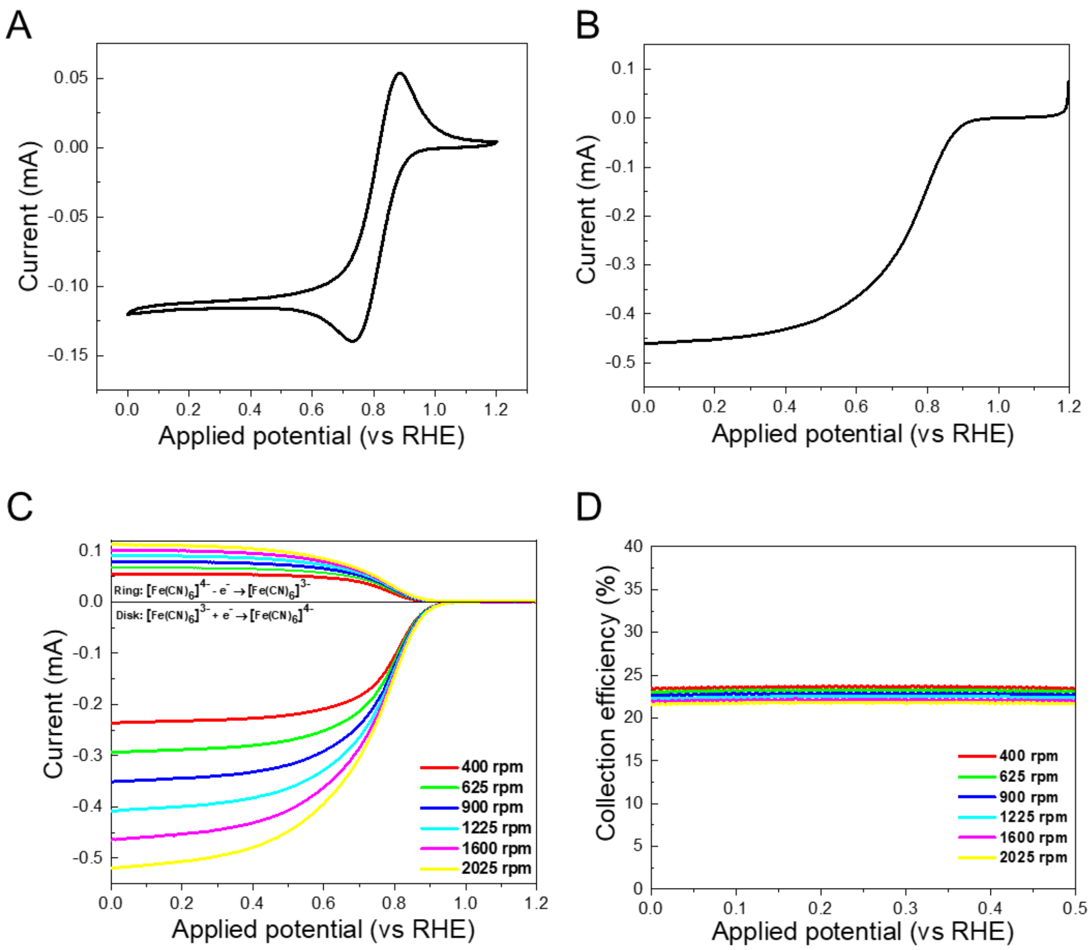

Fig. S20. Calibration of the collection efficiency of the bare RRDE. (**A**) CV scan of the bare GCE disk using a scan rate of 100 mV s^{-1} and 0 rpm in 0.05 M Na_2SO_3 and 0.004 M $K_3[Fe(CN)_6]$ electrolyte. (**B**) LSV curve of PT ring using a scan rate of 50 mV s^{-1} and 1600 rpm in 0.05 M Na_2SO_3 and 0.004 M $K_3[Fe(CN)_6]$ electrolyte. No potential was applied to the GCE electrode. (**C**) RRDE measurements using a scan rate of 50 mV s^{-1} and the ring potential of 1.2 V_{RHE}. (**D**) The calculated collection efficiency at different applied potential.

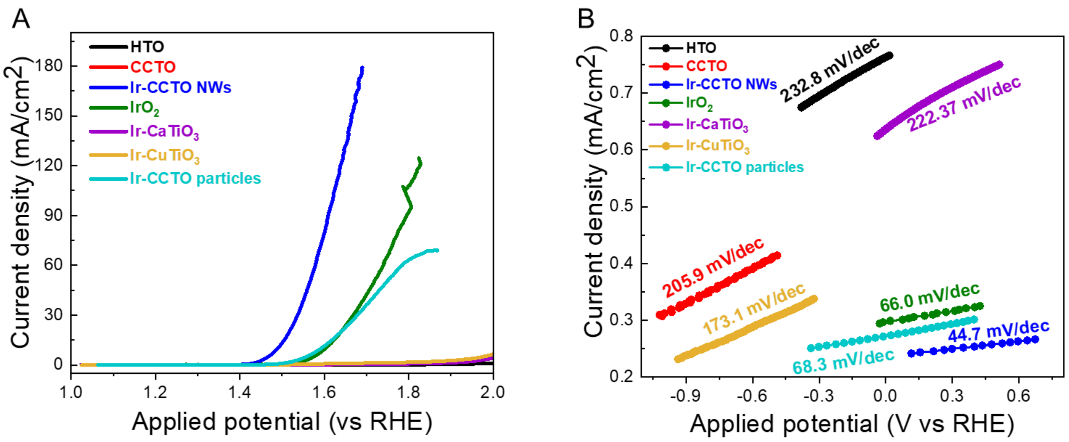

Fig. S21. Comparision of (**A**) LSV plorization curves and (**B**) Tafel slopes of Ir-CCTO nanobelts with various control samples including Ir-$CuTiO_3$, Ir-$CaTiO_3$, and Ir-CCTO nanoparticles.

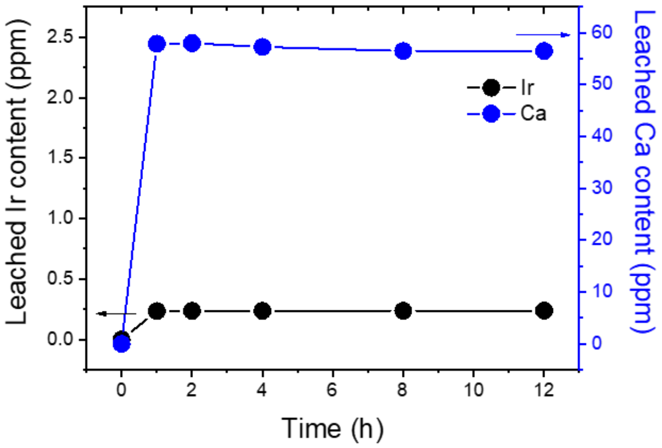

Fig. S22. ICP-OES reuslts for Ir-CCTO during 12 hrs CP measurement.

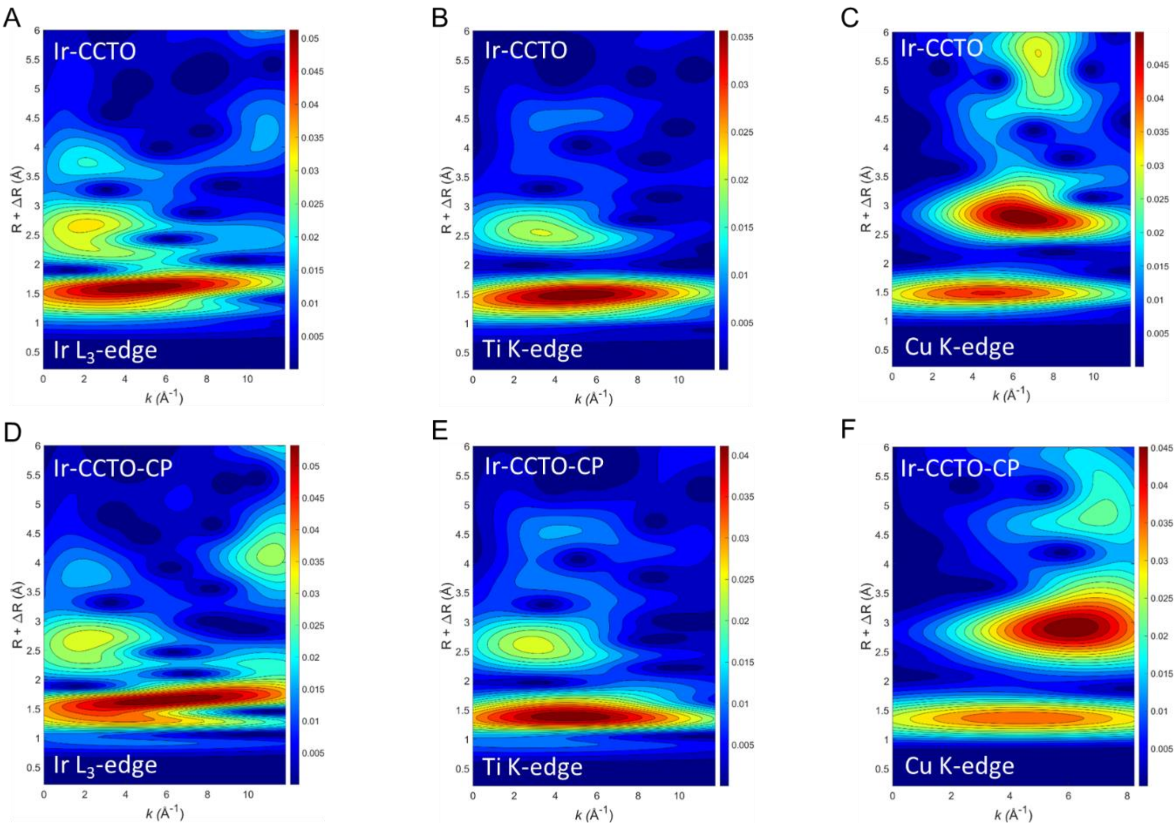

Fig. S23. CCWT plots of Ir L_3-edge, Ti K-edge, and Cu K-edge for (**A-C**) Ir-CCTO and (**D-F**) Ir-CCTO-CP.

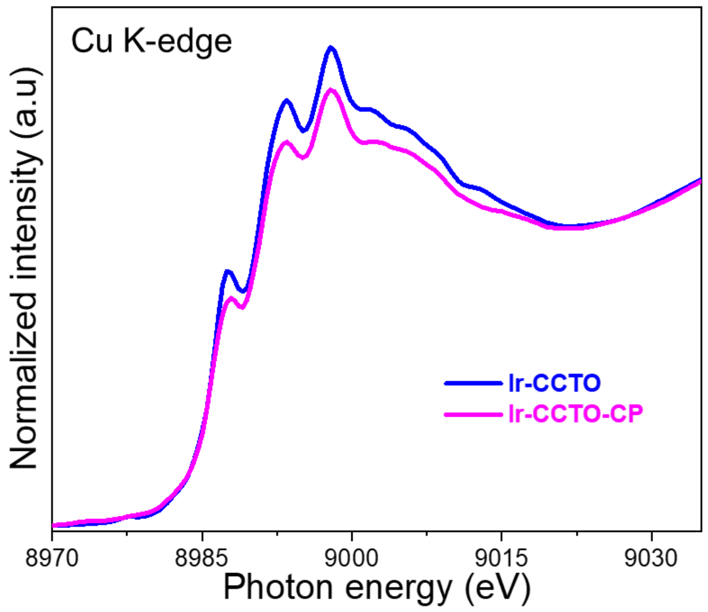

Fig. S24. XANES spectra of Cu K-edge for Ir-CCTO and Ir-CCTO-CP.

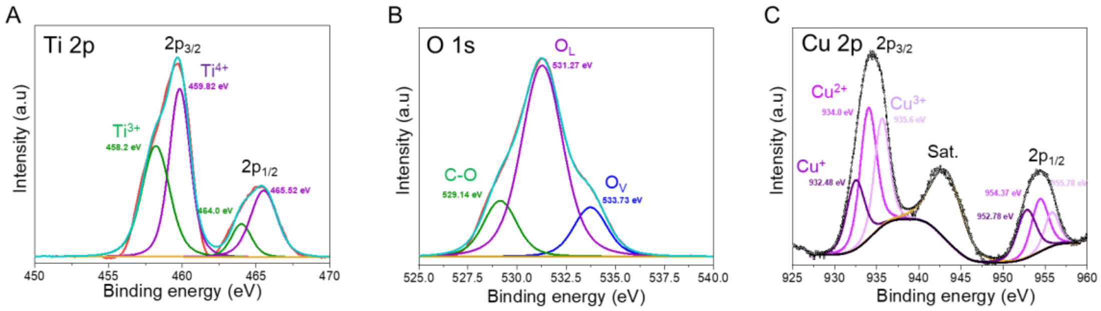

Fig. S25. XPS spectra of (A) Ti 2p, (B) O 1s, and (C) Cu 2p for Ir-CCTO-CP.

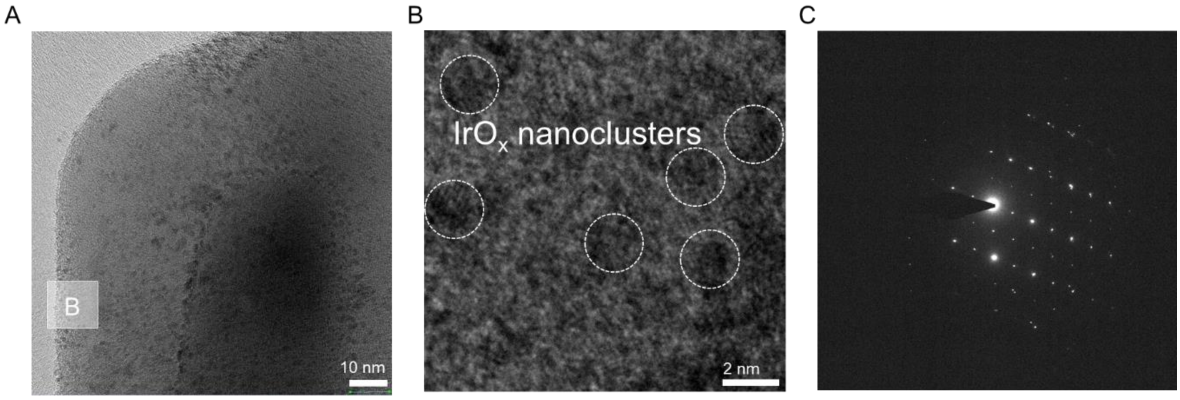

Fig. S26. (**A-B**) HR-TEM images of Ir-CCTO-CP and (**C**) the corresponding SAED image.

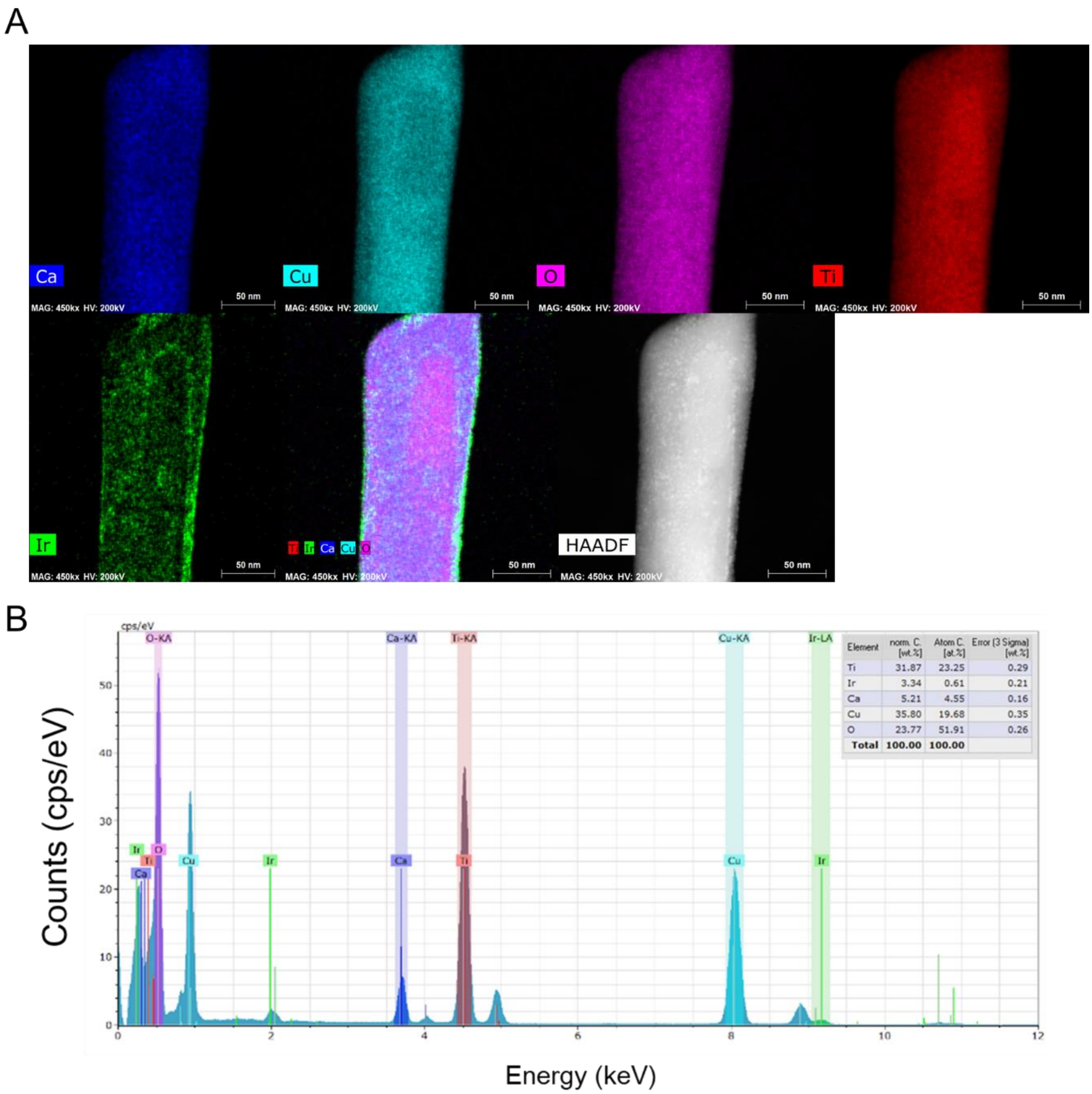

Fig. S27. (**A**) STEM-EDX elemental mapping of Ca, Cu, O, Ti, and Ir for Ir-CCTO-CP and (**B**) the corresponding quantitative spectrum.

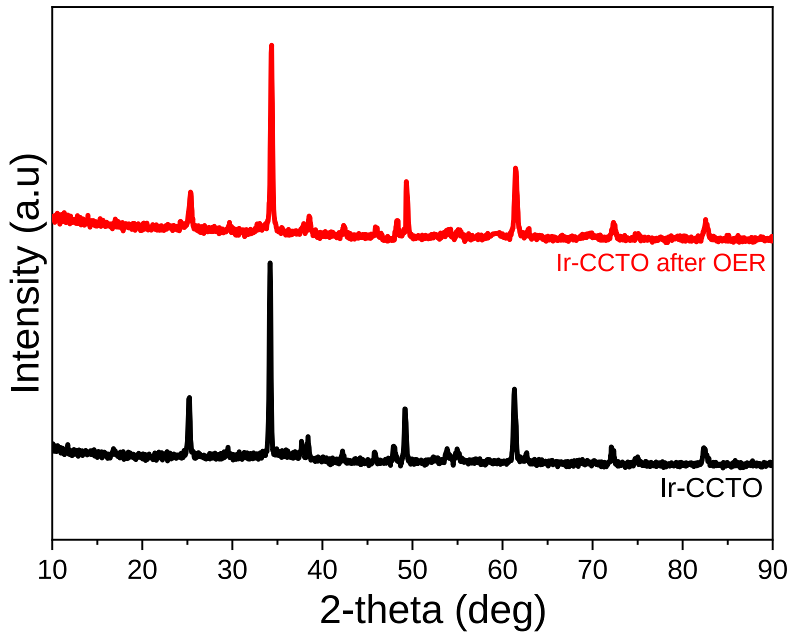

Fig. S28. XRD pattern of Ir-CCTO before and after OER stability test.

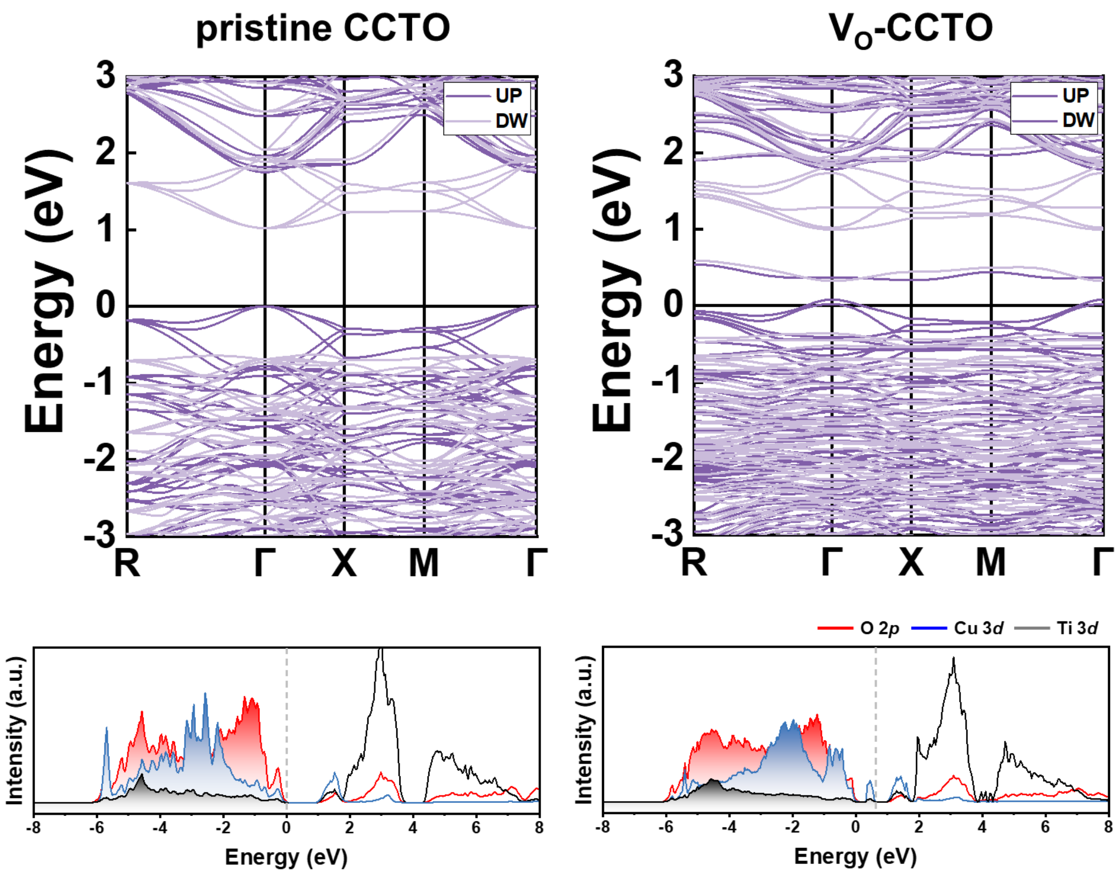

Fig. S29. Band structures and projected density of states (PDOS) for pristine CCTO and V_O-CCTO. The valence band maximum (VBM) for the pristine and stoichiometric CCTO is set to 0 eV, and the band structure and PDOS diagram for V_O-CCTO were then aligned with respect to the core levels of the O atoms at the center of the systems. Dashed lines in PDOS diagrams indicate the VBM.

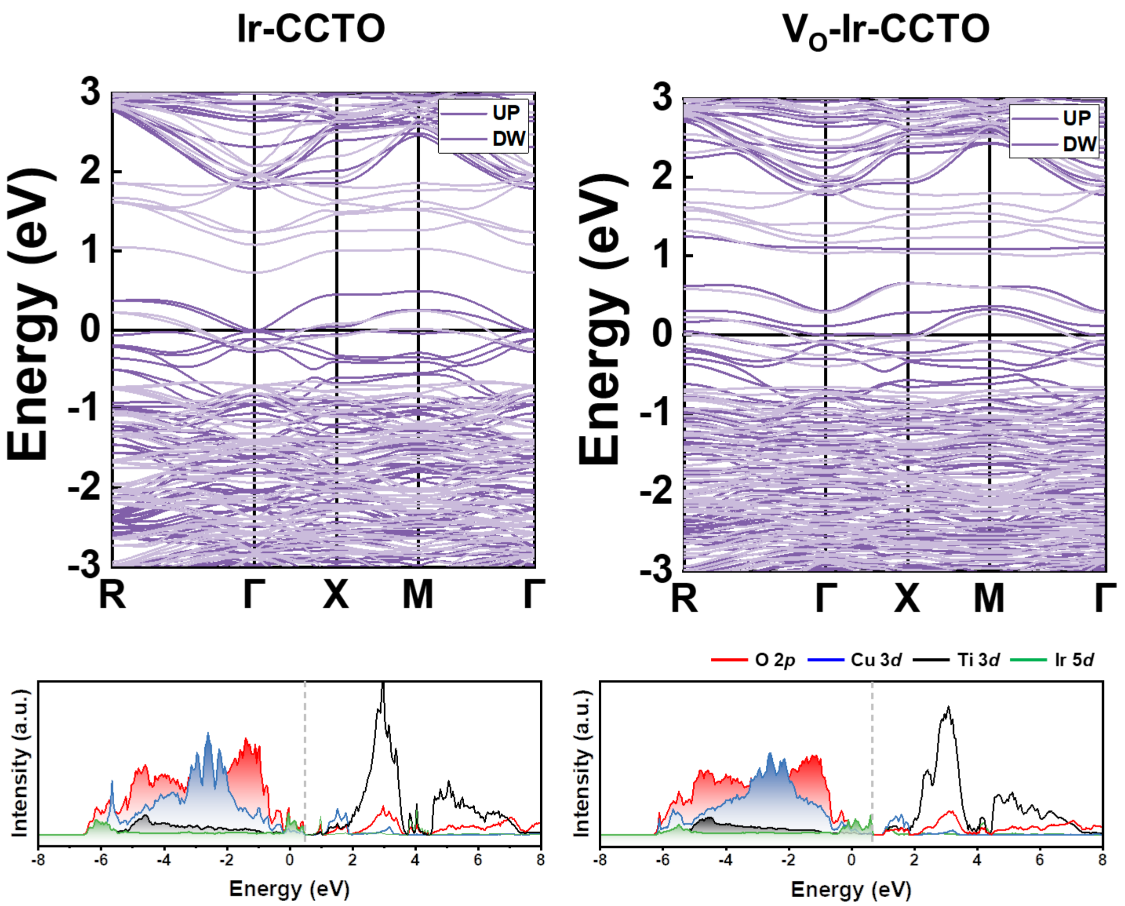

Fig. S30. Band structures and projected density of states (PDOS) for Ir-CCTO and V_O-Ir-CCTO. The valence band maximum (VBM) for the pristine and stoichiometric CCTO is set to 0 eV, and the band structure and PDOS diagram for Ir-CCTO and V_O-Ir-CCTO were then aligned with respect to the core levels of the O atoms at the center of the systems. Dashed lines in PDOS diagrams indicate the VBM.

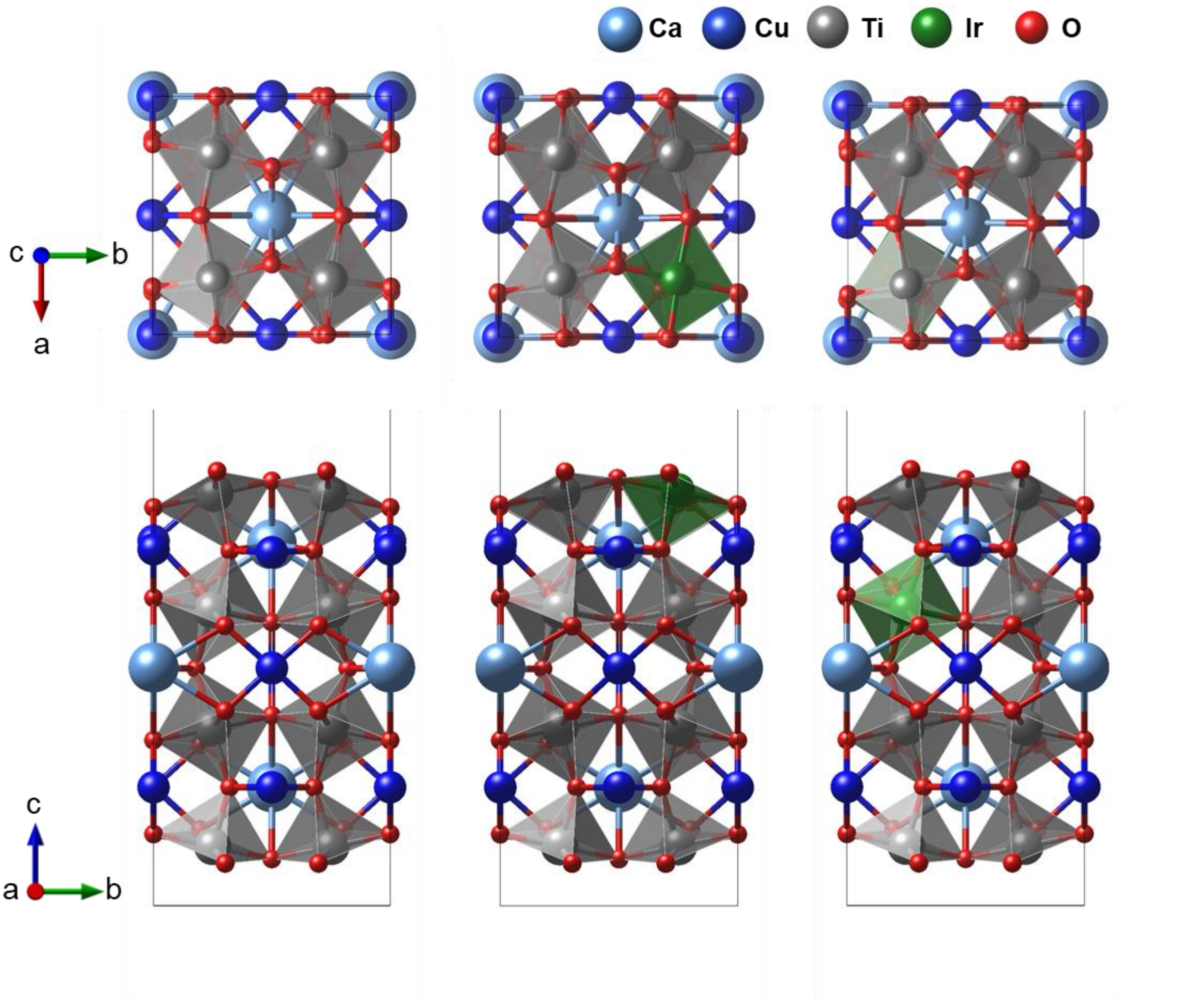

Fig. S31. Geometry-optimized structures for pristine CCTO (001) surface and Ir-doped CCTO (001) surfaces where Ir is doped at the surface and the second layer of the surface.

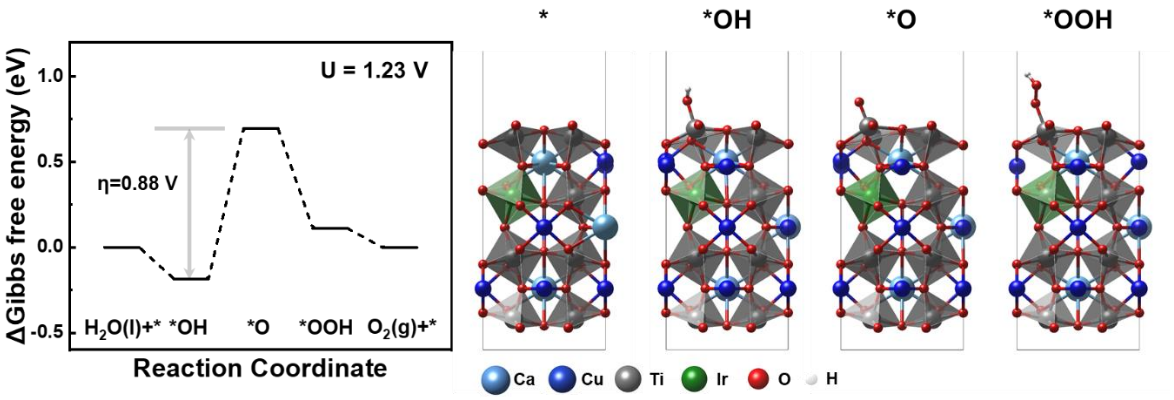

Fig. S32. Calculated Gibbs free energy diagram of OER pathway for Ir-CCTO (001) where Ir is doped at the second layer of the system and its corresponding geometry-optimized atomic configurations**.**

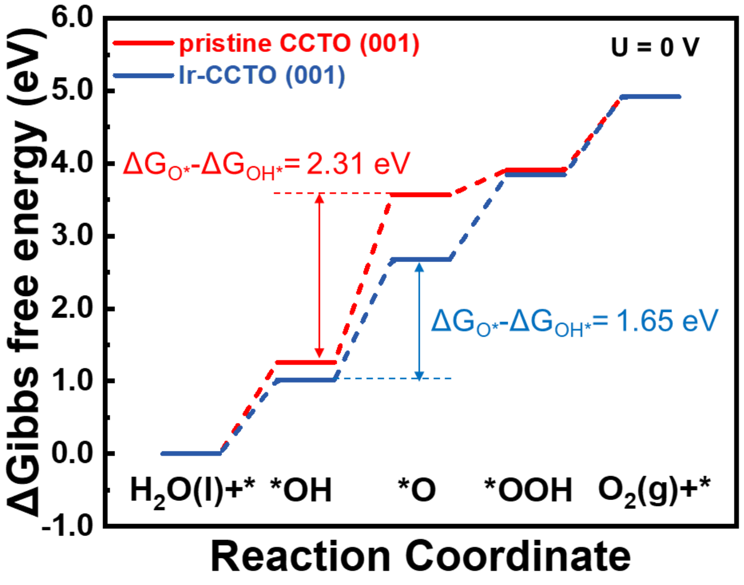

Fig. S33. Calculated Gibbs free energy diagram of OER pathway for CCTO and Ir-CCTO (001) where Ir is doped at the second layer of the system at U=0 V.

Table S1. XPS results of the CCTO, Ir-CCTO and Ir-CCTO CP samples. Peaks are calibrated with reference Carbon 1s.

Samples		Position (eV)	Area (a.u.)	Atomic percentage (%)
Ti^{4+} $2p_{3/2}$ (eV)/(at.%)	CCTO	459	123405.5	61.4
	Ir-CCTO	459.4	108217.9	58.2
	Ir-CCTO-CP	459.8	97466.1	53.3
Ti^{3+} $2p_{3/2}$ (eV)/(at.%)	CCTO	458.3	77676.9	38.6
	Ir-CCTO	458.6	58552.5	41.8
	Ir-CCTO-CP	458.2	85265.6	46.7
Cu^{+} $2p_{3/2}$ (eV)/(at.%)	CCTO	932.4	14189.2	24.2
	Ir-CCTO	933.0	22266.4	31.0
	Ir-CCTO-CP	932.5	18483.2	24.8
Cu^{2+} $2p_{3/2}$ (eV)/(at.%)	CCTO	934.2	36568.5	62.4
	Ir-CCTO	934.8	44278.6	61.7
	Ir-CCTO-CP	934.0	31911.1	42.8
Cu^{3+} $2p_{3/2}$ (eV)/(at.%)	CCTO	935.7	7819.8	13.4
	Ir-CCTO	936.6	5227.7	7.3
	Ir-CCTO-CP	935.6	24153.2	32.4
Ir^{3+} $4f_{7/2}$ (eV)/(at.%)	Ir-CCTO	62.80	8575.5	25.4
	Ir-CCTO-CP	62.13	11072.4	26.4

Ir^{4+} 4$f_{7/2}$ (eV)/(at.%)	Ir-CCTO	61.60	25128.1	74.6
	Ir-CCTO-CP	60.49	30833.7	73.6
O^{2-} 1s (eV)/(at.%)	CCTO	O_L 529.28	319697.9	72.0
		O_V 531.78	124151.7	28.0
	Ir-CCTO	O_L 530.08	238795.5	62.6
		O_V 532.18	143000.4	37.4
	Ir-CCTO-CP	O_L 531.27	71895.5	69.4
		O_V 533.73	307509.5	16.23
		C-O 529.14	63686.4	14.37

Table S2. Structural parameters obtained from the fitting of Ir L3-edge EXAFS spectra

Material	Path	Coordination number	ΔE (eV)	Bond length (Å)	Debye-Waller factor ($\sigma^2 \times 10^{-3}$) Å^2
Ir-CCTO	Ir-O	6	0.736 ± 0.4	1.995 (±0.053)	1.3 (3)
	Ir-Cu	2	0.736 ± 0.4	3.238 (±0.081)	2.1 (2)
	Ir-Ca	2	0.736 ± 0.4	3.351 (±0.091)	3.2 (3)
	Ir-Ti	4	0.736 ± 0.4	3.716 (±0.011)	2.3 (4)
	Ir-Ti-O	8	0.736 ± 0.4	3.879 (±0.021)	3.8 (7)

Table S3. Comparison of OER performance of Ir-CCTO NBs for acidic OER with recently reported catalysts. References for Fig. 4C.

Sample Name	Reference
Rutile RuO_2	*Adv. Energy Mater.* **2019**, *9*, 1803795.
IrOOH nanosheets	*J. Mater. Chem. A* **2018**, *6*, 21558.
IrOOH bulk	*J. Mater. Chem. A* **2018**, *6*, 21558.
Rutile-IrO_2 bulk	*J. Mater. Chem. A* **2018**, *6*, 21558.
Ir-$SrTiO_3$	*Angew. Chem. Int. Ed.* **2019**, *58*, 7631.
Sr_2NiIrO_6	*J. Mater. Chem. A* **2021**, *9*, 2980.
Sr_2CoIrO_6	*J. Mater. Chem. A* **2021**, *9*, 2980.
$Pb_2Ir_2O_{6.5}$	*Sci. Rep.* **2016**, *6*, 38429.
$Ir_{0.06}Co_{22.94}O_4$	*J. Am. Chem. Soc.* **2021**, *143*, 5201.
IrCoNi PHNC	*Adv. Mater.* **2017**, *29*, 1703798.
IrW	*ACS Cent. Sci.* **2018**, *4*, 1244.
ACIN-HF	*Nanoscale Horiz.* **2019**, *4*, 727.
Ru@IrO_x core-shell	*Chem* **2019**, *5*, 445.
SrTi(Ir)O_3	*Angew. Chem. Int. Ed.* **2020**, *59*, 19654.
ICS NC	*Nanoscale* **2020**, *12*, 17074.
Co-doped 6H-SrI_O3	*ACS Appl. Mater. Interfaces* **2019**, *11*, 42006.
6H-$SrIrO_3$	*ACS Appl. Mater. Interfaces* **2019**, *11*, 42006.
Co-RuIr	*Adv. Mater.* **2019**, *31*, 1900510.
IrO_x/9R-$BaIrO_3$	*J. Am. Chem. Soc.* **2021**, *143*, 18001.

Table S4. Ir content in the Ir-CCTO NBs analyzed by the ICP-OES.

atom	Ir
Relative amount (wt.%)	4.21

Table S5. Comparison of mass acitivity of Ir-CCTO NBs for acidic OER with recently reported catalysts. References for Fig. 4F.

Sample Name	Reference
Ir/$TiON_x$/C	*ACS Catal.* **2021**, *11*, 12510.
Ir/$CuTiON_x$/C	*ACS Catal.* **2021**, *11*, 12510.
IrNiCu DNF/C	*Adv. Mater.* **2017**, *29*, 1703798.
SZIO	*Adv. Mater.* **2020**, *32*, 2001430
Ir-STO	*Angew. Chem. Int. Ed.* **2019**, *58*, 7631.
$SrTi(Ir)O_3$	*Angew. Chem. Int. Ed.* **2020**, *59*, 19654.
$Pr_2Ir_2O_7$	*Adv. Mater.* **2019**, *31*, 1805104.
SIO	*Adv. Mater.* **2020**, *32*, 2001430.
Co-doped 6H-$SrIrO_3$	*ACS Appl. Mater. Interfaces* **2019**, *11*, 42006.
IrNi-200-CL	*ACS Catal.* **2018**, *8*, 10498.
$IrNiO_x$	*Nat. Catal.* **2018**, *1*, 841.
IrO_2-TiO_2	*ACS Catal.* **2017**, *7*, 2346.
6H-$SrIrO_3$	*Nat. Commun.* **2018**, *9*, 5236.

Table S6. Comparison table of durability decay for Ir-based catalysts as acidic OER.

Sample	Stability	Reference
Ir-CCTO	50h @ 20 mA/cm^2	Our work
$SrTi_{0.67}IrO_{0.33}O_3$	20h @ 10 mA/cm^2	*Angewandte Chemie*, vol. 131, no. 23, pp. 7713–7717, 2019, doi: 10.1002/ange.201900796.
$SrCo_{0.9}Ir_{0.1}O_{3-\gamma}$	180min @ 10 mA/cm^2	*Nat Commun*, vol. 10, no. 1, Art. no. 1, Feb. 2019, doi: 10.1038/s41467-019-08532-3
IrCoNi PHNCs	200min @ 5mA/cm^2	*Advanced Materials*, vol. 29, no. 47, p. 1703798, 2017, doi: 10.1002/adma.201703798.
IrNi NCs	2h @ 5mA/cm^2	*Advanced Functional Materials*, vol. 27, no. 27, p. 1700886, 2017, doi: 10.1002/adfm.201700886.
$Pr_2Ir_2O_7$	10 000s @ 10 mAcm/disk^2	*Advanced Materials*, vol. 31, no. 6, p. 1805104, 2019, doi: 10.1002/adma.201805104.
$Y_2Ir_2O_7$	24h @ 10mA/cm^2	*ACS Appl. Energy Mater.*, vol. 1, no. 8, pp. 3992–3998, 2018, doi: 10.1021/acsaem.8b00691.
Pd@Ir3L	2000 CV cycles between 1.2 and 1.6 V RHE	*Chem. Mater.*, vol. 31, no. 15, pp. 5867–5875, Aug. 2019, doi: 10.1021/acs.chemmater.9b02011.
IrCo@IrO_x-3L NDs	10h @ 2.5mA/cm^2	*Advanced Materials*, vol. 31, no. 37, p. 1903616, 2019, doi: 10.1002/adma.201903616.
Ru@IrO_x core–shell nanocrystal	24h @ 1.55VRHE	*Chem*, vol. 5, no. 2, pp. 445–459, Feb. 2019, doi: 10.1016/j.chempr.2018.11.010.
$IrCo_{0.65}$ NDs	20 000s @ 10mA/cm^2	*ACS Appl. Mater. Interfaces*, vol. 10, no. 30, pp. 24993–24998, Aug. 2018, doi: 10.1021/acsami.8b08717.
P-$IrCu_{1.4}$ NCs	10h @ 10mA/cm^2	*Chem. Mater.*, vol. 30, no. 23, pp. 8571–8578, Dec. 2018, doi: 10.1021/acs.chemmater.8b03620.
Co-RuIr alloy	25h @ 10 mA/cm^2	*Advanced Materials*, vol. 28, no. 42, pp. 9266–9291, 2016, doi: 10.1002/adma.201602270.
Ir wavy nanowires	25 000s @ 10mA/cm^2	*Nanoscale*, vol. 10, no. 4, pp. 1892–1897, Jan. 2018, doi: 10.1039/C7NR09377B.

$Ir_{44}Pd_{10}$ nanocages	15h @ 10 mA/cm^2	*Angewandte Chemie International Edition*, vol. 58, no. 22, pp. 7244–7248, 2019, doi: 10.1002/anie.201901732.
single-layer IrOOH nanosheets	14h @ 1mA/cm^2	*J. Mater. Chem. A*, vol. 6, no. 43, pp. 21558–21566, Nov. 2018, doi: 10.1039/C8TA07950A.
6H-$SIrO_3$	30h @ 10 mA/cm^2	*Nat Commun*, vol. 9, no. 1, Art. no. 1, Dec. 2018, doi: 10.1038/s41467-018-07678-w.
H_2IrO_3	50h @ 10 mA/cm^2	*Chem. Mater.*, vol. 31, no. 15, pp. 5845–5855, Aug. 2019, doi: 10.1021/acs.chemmater.9b01976.
La_3IrO_7	24h @ 10 mA/cm^2	*Chem. Mater.*, vol. 29, no. 12, pp. 5182–5191, Jun. 2017, doi: 10.1021/acs.chemmater.7b00766.
$IrO_x/SrIrO_3$	30h @ 10 mA/cm^2	*Science*, vol. 353, no. 6303, pp. 1011–1014, Sep. 2016, doi: 10.1126/science.aaf5050.
9R-$BaIrO_3$	48h @10 mA/cm^2	*J. Am. Chem. Soc.*, vol. 143, no. 43, pp. 18001–18009, Nov. 2021, doi: 10.1021/jacs.1c04087.

Table S7. Calculated lattice constant and magnetic moment on CuO_4, compared with other studies for pristine and stoichiometric CCTO.

Method	Structure (space group)	Lattice constant (Å)	Magnetic moment on CuO_4 (μ_B)	Ref.
GGA+U	cubic (Im-3)	7.450	0.90	This work
GGA+U	cubic (Im-3)	7.380	·	[14]
GGA	cubic (Im-3)	7.459	·	[25]
GGA	cubic (Im-3)	7.500	~0.85	[26]
GGA	cubic (Im-3)	7.404	0.84	[27]
LSDA	cubic (Im-3)	7.290	0.85	[28]
exp.	cubic (Im-3)	7.393	·	[25]
exp.	cubic (Im-3)	7.391 (at 25℃)	·	[29]
exp.	cubic (Im-3)	7.391	·	[30]

References

1. B. Ravel and M. Newville, ATHENA, ARTEMIS, HEPHAESTUS: data analysis for X-ray absorption spectroscopy using IFEFFIT, *J Synchrotron Radiat*, 12, 537-541 (2005).
2. M. Munoz, P. Argoul and F. Farges, Continuous Cauchy wavelet transform analyses of EXAFS spectra: A qualitative approach, *Am Mineral*, 88, 694-700 (2003).
3. M. A. Lukowski, A. S. Daniel, C. R. English, F. Meng, A. Forticaux, R. J. Hamers and S. Jin, Highly active hydrogen evolution catalysis from metallic WS_2 nanosheets, *Energ Environ Sci*, 7, 2608-2613 (2014).
4. Y. R. Hong, K. M. Kim, J. H. Ryu, S. Mhin, J. Kim, G. Ali, K. Y. Chung, S. Kang and H. Han, Dual - phase engineering of nickel boride - hydroxide nanoparticles toward high - performance water oxidation electrocatalysts, *Advanced Functional Materials*, 30, 2004330 (2020).
5. H. Han, H. Choi, S. Mhin, Y.-R. Hong, K. M. Kim, J. Kwon, G. Ali, K. Y. Chung, M. Je, H. N. Umh, D.-H. Lim, K. Davey, S.-Z. Qiao, U. Paik and T. Song, Advantageous crystalline–amorphous phase boundary for enhanced electrochemical water oxidation, *Energy & Environmental Science*, 12, 2443-2454 (2019).
6. Kresse, G., Hafner, J. Ab initio molecular-dynamics simulation of the liquid-metalamorphous- semiconductor transition in germanium. *Phys. Rev. B* 49, 14251–14269 (1994).
7. Kresse, G., Hafner, J. Ab initio molecular dynamics for liquid metals. *Phys. Rev. B* 47, 558–561 (1993).
8. Kresse, G., Furthmüller, J. Efficiency of ab-initio total energy calculations for metals and semiconductors using a plane-wave basis set. *Comput. Mater. Sci.* 6, 15–50 (1996).
9. Kresse, G., Furthmüller, J. Efficient iterative schemes for ab initio total-energy calculations using a plane-wave basis set. *Phys. Rev. B* 54, 11169–11186 (1996).
10. Blöchl, P. E. Projector augmented-wave method. *Phys. Rev. B* 50, 17953–17979 (1994).
11. Kresse, G., Joubert, D. From ultrasoft pseudopotentials to the projector augmented-wave method. *Phys. Rev. B* 59, 1758–1775 (1999).
12. Perdew, J. P., Burke, K. & Ernzerhof, M. Generalized gradient approximation made simple. *Phys. Rev. Lett.* 77, 3865–3868 (1996).
13. Dudarev, S., Botton, G. Electron-energy-loss spectra and the structural stability of nickel oxide: An LSDA+U study. *Phys. Rev. B - Condens. Matter Mater. Phys.* 57, 1505–1509 (1998).
14. Alippi, P., Fiorentini, V. Magnetism and unusual Cu valency in quadruple perovskites. *Eur. Phys. J. B* 85, (2012).
15. Panda, S. K., Bhowal, S., Delin, A., Eriksson, O. & Dasgupta, I. Effect of spin orbit coupling and Hubbard U on the electronic structure of IrO_2. *Phys. Rev. B - Condens. Matter Mater. Phys.* 89, 1–7 (2014).
16. Monkhorst, Hendrik J., Pack, J. D. Special points for Brillonin-zone integrations. *Phys. Rev. B* 13, 5188–5192 (1976).
17. Wang, Y. & Cheng, H. P. Oxygen reduction activity on perovskite oxide surfaces: A comparative first-principles study of $LaMnO_3$, $LaFeO_3$, and $LaCrO_3$. *J. Phys. Chem. C* 117, 2106–2112 (2013).
18. Eglitis, R. I. Comparative first-principles calculations of SrTiO3, BaTiO3, $PbTiO_3$ and $CaTiO_3$ (001), (011) and (111) surfaces. *Ferroelectrics* 483, 53–67 (2015).
19. Guo, Y. *et al.* Effects of Rb Incorporation and Water Degradation on the Stability of the Cubic Formamidinium Lead Iodide Perovskite Surface: A First-Principles Study. *J. Phys. Chem. C* 121, 12711–12717 (2017).

20. Eglitis, R. & Kruchinin, S. P. Ab initio calculations of ABO perovskite (001), (011) and (111) nano-surfaces, interfaces and defects. *Mod. Phys. Lett. B* 34, 2040057 (2020).
21. Suntivich, J. *et al.* Design principles for oxygen-reduction activity on perovskite oxide catalysts for fuel cells and metal-air batteries. *Nat. Chem.* 3, 546–550 (2011).
22. Zhao, B. *et al.* A tailored double perovskite nanofiber catalyst enables ultrafast oxygen evolution. *Nat. Commun.* 8, 1–9 (2017).
23 Nørskov, J. K. *et al.* Origin of the overpotential for oxygen reduction at a fuel-cell cathode. *J. Phys. Chem. B* 108, 17886–17892 (2004).
24 Gauthier, J. A., Dickens, C. F., Chen, L. D., Doyle, A. D. & Nørskov, J. K. Solvation Effects for Oxygen Evolution Reaction Catalysis on IrO2(110). *J. Phys. Chem. C* 121, 11455–11463 (2017).
25. Boonlakhorn, J., Chanlek, N., Thongbai, P. & Srepusharawoot, P. Strongly Enhanced Dielectric Response and Structural Investigation of (Sr^{2+}, Ge^{4+}) Co-Doped CCTO Ceramics. *J. Phys. Chem. C* 124, 20682–20692 (2020).
26. Alippi, P., Fiorentini, V. & Filippetti, A. Electronic Structure of Bulk and Defected $CaCu_3Ti_4O_{12}$. *ECS Trans.* 3, 291–297 (2006).
27. Li, G. L., Yin, Z. & Zhang, M. S. First-principles study of the electronic and magnetic structures of $CaCu_3Ti_4O_{12}$. *Phys. Lett. Sect. A Gen. At. Solid State Phys.* 344, 238–246 (2005).
28. He, L., Neaton, J. B., Cohen, M. H., Vanderbilt, D. & Homes, C. C. First-principles study of the structure and lattice dielectric response of CaCu3Ti4O12. *Phys. Rev. B - Condens. Matter Mater. Phys.* 65, 2141121–21411211 (2002).
29. Subramanian, M. A., Li, D., Duan, N., Reisner, B. A. & Sleight, A. W. High dielectric constant in ACu3Ti4O12 and ACu3Ti3FeO12 phases. *J. Solid State Chem.* 151, 323–325 (2000).
30. Bochu, B. *et al*. Synthèse et caractérisation d'une série de titanates pérowskites isotypes de $[CaCu_3](Mn_4)O_{12}$. *J. Solid State Chem.* 29, 291–298 (1979).
31. X. Liang et al., 'Activating Inert, Nonprecious Perovskites with Iridium Dopants for Efficient Oxygen Evolution Reaction under Acidic Conditions', *Angewandte Chemie*, vol. 131, no. 23, pp. 7713–7717, 2019, doi: 10.1002/ange.201900796.
32. Y. Chen et al., 'Exceptionally active iridium evolved from a pseudo-cubic perovskite for oxygen evolution in acid', *Nat Commun*, vol. 10, no. 1, Art. no. 1, Feb. 2019, doi: 10.1038/s41467-019-08532-3.
33. J. Feng et al., 'Iridium-Based Multimetallic Porous Hollow Nanocrystals for Efficient Overall-Water-Splitting Catalysis', *Advanced Materials*, vol. 29, no. 47, p. 1703798, 2017, doi: 10.1002/adma.201703798.
34. Y. Pi, Q. Shao, P. Wang, J. Guo, and X. Huang, 'General Formation of Monodisperse IrM (M = Ni, Co, Fe) Bimetallic Nanoclusters as Bifunctional Electrocatalysts for Acidic Overall Water Splitting', *Advanced Functional Materials*, vol. 27, no. 27, p. 1700886, 2017, doi: 10.1002/adfm.201700886.
35. C. Shang et al., 'Electron Correlations Engineer Catalytic Activity of Pyrochlore Iridates for Acidic Water Oxidation', *Advanced Materials*, vol. 31, no. 6, p. 1805104, 2019, doi: 10.1002/adma.201805104.
36. P.-C. Shih, J. Kim, C.-J. Sun, and H. Yang, 'Single-Phase Pyrochlore Y2Ir2O7 Electrocatalyst on the Activity of Oxygen Evolution Reaction', *ACS Appl. Energy Mater.*, vol. 1, no. 8, pp. 3992–3998, Aug. 2018, doi: 10.1021/acsaem.8b00691.
37. J. Zhu et al., 'Facile Synthesis and Characterization of Pd@IrnL (n = 1–4) Core–Shell Nanocubes for Highly Efficient Oxygen Evolution in Acidic Media', *Chem. Mater.*, vol. 31, no. 15, pp. 5867–5875, Aug. 2019, doi: 10.1021/acs.chemmater.9b02011.

38. G. Meng et al., ‘Strain Regulation to Optimize the Acidic Water Oxidation Performance of Atomic-Layer IrOx’, *Advanced Materials*, vol. 31, no. 37, p. 1903616, 2019, doi: 10.1002/adma.201903616.
39. J. Shan et al., ‘Charge-Redistribution-Enhanced Nanocrystalline Ru@IrOx Electrocatalysts for Oxygen Evolution in Acidic Media’, Chem, vol. 5, no. 2, pp. 445–459, Feb. 2019, doi: 10.1016/j.chempr.2018.11.010.
40. L. Fu, X. Zeng, G. Cheng, and W. Luo, ‘IrCo Nanodendrite as an Efficient Bifunctional Electrocatalyst for Overall Water Splitting under Acidic Conditions’, *ACS Appl. Mater. Interfaces*, vol. 10, no. 30, pp. 24993–24998, Aug. 2018, doi: 10.1021/acsami.8b08717.
41. Y. Pi, J. Guo, Q. Shao, and X. Huang, ‘Highly Efficient Acidic Oxygen Evolution Electrocatalysis Enabled by Porous Ir–Cu Nanocrystals with Three-Dimensional Electrocatalytic Surfaces’, *Chem. Mater.*, vol. 30, no. 23, pp. 8571–8578, Dec. 2018, doi: 10.1021/acs.chemmater.8b03620.
42. L. Han, S. Dong, and E. Wang, ‘Transition-Metal (Co, Ni, and Fe)-Based Electrocatalysts for the Water Oxidation Reaction’, *Advanced Materials*, vol. 28, no. 42, pp. 9266–9291, 2016, doi: 10.1002/adma.201602270.
43. L. Fu, F. Yang, G. Cheng, and W. Luo, ‘Ultrathin Ir nanowires as high-performance electrocatalysts for efficient water splitting in acidic media’, *Nanoscale*, vol. 10, no. 4, pp. 1892–1897, Jan. 2018, doi: 10.1039/C7NR09377B.
44. J. Zhu et al., ‘Iridium-Based Cubic Nanocages with 1.1-nm-Thick Walls: A Highly Efficient and Durable Electrocatalyst for Water Oxidation in an Acidic Medium’, *Angewandte Chemie International Edition*, vol. 58, no. 22, pp. 7244–7248, 2019, doi: 10.1002/anie.201901732.
45. D. Weber et al., ‘IrOOH nanosheets as acid stable electrocatalysts for the oxygen evolution reaction’, *J. Mater. Chem. A*, vol. 6, no. 43, pp. 21558–21566, Nov. 2018, doi: 10.1039/C8TA07950A.
46. L. Yang et al., ‘Efficient oxygen evolution electrocatalysis in acid by a perovskite with face-sharing IrO6 octahedral dimers’, *Nat Commun*, vol. 9, no. 1, Art. no. 1, Dec. 2018, doi: 10.1038/s41467-018-07678-w.
47. P. E. Pearce et al., ‘Revealing the Reactivity of the Iridium Trioxide Intermediate for the Oxygen Evolution Reaction in Acidic Media’, *Chem. Mater.*, vol. 31, no. 15, pp. 5845–5855, Aug. 2019, doi: 10.1021/acs.chemmater.9b01976.
48. D. Lebedev et al., ‘Highly Active and Stable Iridium Pyrochlores for Oxygen Evolution Reaction’, *Chem. Mater.*, vol. 29, no. 12, pp. 5182–5191, Jun. 2017, doi: 10.1021/acs.chemmater.7b00766.
49. L. C. Seitz et al., ‘A highly active and stable IrOx/SrIrO3 catalyst for the oxygen evolution reaction’, *Science*, vol. 353, no. 6303, pp. 1011–1014, Sep. 2016, doi: 10.1126/science.aaf5050.
50. N. Li et al., ‘Identification of the Active-Layer Structures for Acidic Oxygen Evolution from 9R-BaIrO3 Electrocatalyst with Enhanced Iridium Mass Activity’, *J. Am. Chem. Soc.*, vol. 143, no. 43, pp. 18001–18009, Nov. 2021, doi: 10.1021/jacs.1c04087.
